# Deciphering of intra‐tumoural heterogeneity and the interplay between metastasis‐associated meta‐program and myofibroblasts in gastric cancer

**DOI:** 10.1002/ctm2.70319

**Published:** 2025-04-28

**Authors:** Xiongyan Wu, Zhijian Jin, Baolong Li, Yifan Lu, Junyi Hou, Lizhong Yao, Zhenjia Yu, Qingqing Sang, Beiqin Yu, Jianfang Li, Chen Li, Chao Yan, Zhenggang Zhu, Kaiwen Tang, Bingya Liu, Liping Su

**Affiliations:** ^1^ Department of General Surgery Shanghai Key Laboratory of Gastric Neoplasms Shanghai Institute of Digestive Surgery Ruijin Hospital Shanghai Jiao Tong University School of Medicine Shanghai China

**Keywords:** gastric cancer, intra‐tumoural heterogeneity, myofibroblasts, tumour immune microenvironment

## Abstract

**Background:**

Gastric cancer (GC) exhibits high heterogeneity that relies on the oncogenic properties of cancer cells and multicellular interactions in the tumour microenvironment. However, the heterogeneity of GC and their molecular characteristics are still largely unexplored.

**Methods:**

We employed single‐cell and spatial transcriptomics to comprehensively map the intra‐tumoural heterogeneity within GC. Additionally, in vitro experiments, clinical sample analyses, and patient‐derived organoid models (PDOs) were conducted to validate the key interaction patterns between tumor cells and stromal cells.

**Results:**

Seven robust meta‐programs (MP1–MP7) in GC were defined with distinct biological significance and spatial distributions. MP3 and MP4 were intimately associated with distinct CD8 T cells skewed toward a cytotoxic or exhaustion state, while MP7, characterised by the highest degree of malignancy, harboured an immune lockdown microenvironment around it and spatially associated with myofibroblasts (myCAFs). Notably, we clarified the interplay between the MP7 and myCAFs, where MP7 induces the chemotactic migration of fibroblasts and promoting their transformation into myCAFs via GDF15/TGFBR2, and in turn, myCAFs‐derived RSPO3 up‐regulates EGR1 to promote the transformation to MP7 in GC cells and human PDOs. Ultimately, the accumulation of myCAFs around MP7 led to fewer infiltration of CD8 T cells, resulting an immune‐deprived microenvironment and the diminished efficacy of immunotherapy. Additionally, based on the gene expression signatures of MP7 GC cells, we predicted specific drugs and verified more potent inhibitory effects of Taselisib and Lapatinib for MP7 GC cells than conventional drugs at the same concentration.

**Conclusion:**

Taken together, these results deepened the understanding of GC heterogeneity and paved the way for novel therapeutic strategies by targeting MP7 GC cells and their interaction loop with myCAFs in GC treatment.

**Key points:**

- Seven robust meta‐programs (MP1‐MP7) were identified in gastric cancer.
- MP7 was strongly correlated with cancer metastasis and poor survival of gastric cancer patients.
- MP7 promoted fibroblast transformation into myCAFs via GDF15/TGFBR2, creating an immune lockdown microenvironment.
- MyCAFs induced MP7 transformation via the RSPO3/EGR1 pathway, promoting gastric cancer cell migration.
- Taselisib and Lapatinib were potent inhibitors of MP7 GC cells.

## Graphical abstract

Seven robust meta‐programs were identified in GC. The crosstalk between MP7 and myCAFs creates an immune lockdown microenvironment and promotes cancer metastasis, resulting in poor survival of GC patients.

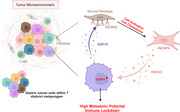

## 1 INTRODUCTION

Gastric cancer (GC) is the fourth leading cause of cancer‐related mortality worldwide, resulting in 769 000 deaths in 2020.^1^ Although early‐stage GC can be effectively treated via surgical resection, approximately 50% of GC patients are diagnosed at an advanced stage, with a 5‐year survival rate of less than 5% for those with metastatic disease. For advanced GC, surgical resection coupled with perioperative chemotherapy is the primary curative approach.^2^ In addition to adjuvant chemotherapy, targeted therapies, such as inhibitors targeting human epidermal growth factor receptor 2 (HER2) and vascular endothelial growth factor receptor (VEGFR), are also gradually being employed in the treatment of GC.^3^, ^4^ Immune checkpoint blockade has revolutionised the paradigm in clinical oncology. In a recent multi‐centre, randomised phase III trial, the SOXRC regimen, comprising an anti‐PD‐1 antibody, a VEGFR‐2 inhibitor, S‐1 and oxaliplatin, significantly improved pathological complete response rates compared with those of SOX alone (18.3 vs. 5.0%).^5^ However, the responses of patients to these therapies are still limited and highly variable, with unclear underlying reasons.^4^, ^5^, ^6^, ^7^

GC exhibits high heterogeneity in terms of intra‐tumour and inter‐individual variation, leading to an aggressive phenotype, therapeutic resistance and immune evasion.^8^, ^9^, ^10^, ^11^, ^12^ The tumour microenvironment (TME) is a complex ecosystem composed of lymphocytes, myeloid cells, endothelial cells and cancer‐associated fibroblasts (CAFs).^13^, ^14^ The reciprocal communication between these cells influences the genetic and epigenetic landscape of tumours, culminates in a diverse TME and enhances the intra‐tumoural heterogeneity (ITH).^12^, ^15^, ^16^, ^17^, ^18^, ^19^, ^20^, ^21^ Thus, there is an urgent need to understand the TME to identify new therapeutic targets and improve clinical outcomes in patients with GC.

Single‐cell RNA sequencing (scRNA‐seq) has efficiently unveiled the characterisation of ITH and has rapidly expanded its application across a spectrum of common cancer types, including GC.^22^, ^23^, ^24^, ^25^ Although these various cell types and subclusters guide our understanding of tumour heterogeneity, much remains to be elucidated regarding their functional impact on tumour pathophysiology, interaction with the TME and patient outcomes, as well as the mechanisms underlying their formation and plasticity.^26^ The concept of ‘meta‐programs’ (MPs) involves dozens of genes with coordinated expression variability in malignant cells and has been applied to characterise ITH within specific tumours, such as colon cancer, oesophageal squamous cell carcinoma, hepatocellular carcinoma and cervical squamous cell carcinoma.^26^, ^27^, ^28^, ^29^, ^30^ Importantly, specific cell types within the TME have been found to have a causative relationship with cancer cell states.^26^, ^31^, ^32^, ^33^, ^34^ In colorectal cancer research, the proliferation stemness MP was notably enriched in left‐sided malignant epithelia, shaping the glucose metabolism reprogramming niche, whereas the immune secretory MP exhibited specific enrichment in right‐sided malignant epithelia, resulting in an immune escape microenvironment via interactions with macrophages.^28^ However, in GC, the phenotypic heterogeneity of malignant and non‐malignant cells in the TME and their crosstalk, which contributes to initiating, controlling and maintaining the ‘cell‐state’, remain unclear.

Here, we illustrated the heterogeneity of GC and identified seven distinct MPs through integrative analyses, including scRNA‐seq, spatial transcriptome sequencing (ST‐seq), bulk RNA sequencing and multiplex immunofluorescence staining (mIF). We defined seven robust MPs in GC with distinct biological significance and spatial distribution and demonstrated that the MP7 is associated with the poorest prognosis of GC patients and displays the highest metastatic potential. In addition, we revealed that MP7 and myCAFs are closely linked both spatially and through molecular communication mediated by the GDF15/RSPO3 axis, which induces and maintains their state. Finally, we implemented the organoid model in combination with GC cell lines to investigate therapeutic approaches by targeting MP7, providing evidence‐based strategies to improve outcomes for GC patients.

## 2 METHODS

### 2.1 Single‐cell RNA‐seq

Five fresh GC samples were profiled via the BD Rhapsody system to obtain single‐cell transcriptomic data, constituting our in‐house cohort. Two public GC scRNA‐seq datasets were obtained from the GEO database (GSE183904 and GSE231540). The outputs of three cohorts were subsequently imported into the Seurat (v5.0.1) R toolkit for quality control and downstream analysis. In detail, applied for normalisation to adjust for variations in transcript counts, and ‘FindVariableFeatures’ was used to identify highly variable genes that may be related to cellular heterogeneity. ‘ScaleData’ and ‘RunPCA’ were used for dimensionality reduction and clustering, while ‘IntegrateLayers’ and ‘HarmonyIntegration’ were utilised for sample integration and batch effect removal. ‘FindNeighbors’, ‘FindClusters’ and ‘RunUMAP’ were used for clustering and visualisation. ‘FindAllMarkers’ identified characteristic genes, and cell types were defined via the SingleR toolkit and known markers. All functions were run with default parameters, unless specified otherwise.

Data quality control and sample selection involve the following aspects: (1) Cell filtering: low‐quality cells or doublets were excluded using thresholds of nFeature RNA > 300 and nCount RNA < 7500, which are commonly used thresholds to remove cells with insufficient gene expression coverage or excessive ambient RNA, respectively. Moreover, the DoubletFinder R package was utilised to identify doublets and the top 7.5% of cells on the basis of the doublet score were removed. (2) Gene filtering: Genes that were expressed in fewer than 100 cells were eliminated. (3) Sample filtering: After defining the cell types, samples that contained fewer than 200 epithelial cells were excluded. As a result, 44 samples were retained for downstream analysis.

In addition, two datasets, GSE167297 and GSE163558, both obtained from the GEO database, were employed to assess the differences in invasiveness and metastatic potential across various meta programs. Specifically, GSE167297 comprised single‐cell data from five pairs of superficial and deep GC tissues, while GSE163558 encompassed single‐cell data from three primary tumours and six metastatic sites.

### 2.2 Expression programs associated with ITH in GC

To capture GC heterogeneity, we employed non‐negative matrix factorisation algorithm (NMF) on each tumour sample to generate MPs according to the tutorial^26^ (https://github.com/tiroshlab/3ca/tree/main/ITH_hallmarks). First, NMF was run for each tumour with factors (K) ranging from 4 to 9, yielding 39 programs per sample. Each program was defined by the top 50 genes from the NMF coefficients, totalling 1716 programs. Second, robust NMF programs that appeared in more than one NMF rank within the sample and had similarity to an NMF program in a different sample were identified. Three criteria were used: (1) 70% gene overlap with different K programs in the same sample, (2) 20% overlap with any program in other samples and (3) no more than 30% overlap with other programs in the same tumour. This approach yielded a total of 210 robust NMF programs. Third, these robust NMF programs were clustered by Jaccard similarity, selecting programs with at least eight gene overlaps as potential cluster founders. If overlaps exceeded five cases, the program with the highest overlap was added, forming a cluster. The MP was initially defined by common genes and then completed for 50 genes based on NMF scores. This process was repeated, adding programs with at least 10 gene overlaps and updating the MP. Finally, seven GC MPs, each consisting of 50 genes, were emerged.

To assign each tumour cell to a certain MP, a cell score was calculated according to the tutorial.^26^ Briefly, the scRNA expression matrix for each sample underwent normalisation to stabilise variance, and the ‘sigScores’ function from the scalop R package (v1.1.0) was used to score each cell, with at least 25 genes conserved in the MP. Cells were assigned to the MP with the highest score. An MP must be expressed in at least 5% of cells to ensure sample consistency, and cells not meeting these criteria are labelled ‘unresolved’. To quantitatively assess the expression of MPs within individual cells, MP scores were also calculated via the function ‘AddModuleScore’.

### 2.3 Signalling pathway enrichment analysis

To investigate the biological states or functional differences of MPs, Gene Set Variation Analysis (GSVA) was employed for pathway enrichment analysis of the scRNA‐seq data. The enrichment analysis utilised signatures from Molecular Signatures Database (MsigDB), including Gene Ontology (C5‐GO:BP), REACTOME (C2‐CP:REACTOME) and Kyoto Encyclopedia of Genes and Genomes (C2‐CP:KEGG) pathways. GSVA enrichment scores were calculated via the ‘gsvaParam’ and ‘gsva’ functions from the GSVA R package (v1.52.3). Signatures with adjusted *p* values < .05 were deemed significantly enriched.

### 2.4 Inferring MP regulators

To increase the signal‐to‐noise ratio in data and avoid biases from incorrect modelling or over‐smoothing, the metacell algorithm was applied prior to gene regulatory network analysis.^35^ Subsequently, pySCENIC (v0.12.1) was utilised to assess transcriptional factor (TF) activities in meta cells, following the protocol at https://github.com/aertslab/SCENICprotocol.

### 2.5 Spatial transcriptome

Spatial transcriptomics (ST) data used Visium 10× platform were obtained from the GEO database for the GSE251950 cohort (containing nine primary GC tissues). Downstream analysis of STs was performed according to the standard pipeline of Seurat (https://satijalab.org/seurat/articles/spatial_vignette). Spots were annotated using robust cell type decomposition (RCTD) with a reference subset derived from previous GC scRNA‐seq data, which included 12 different cell types (excluding proliferating cell type).^36^ The RCTD was run in ‘Multi mode’, outputting the estimated weights of each cell type within a spot. An alternative method was also used, integrating cell‐type labels from ‘SCTransform’ processed scRNA‐seq data into normalised ST data via the ‘FindTransferAnchors’ and ‘TransferData’ functions. The spots were annotated based on the highest prediction score. Tumour cell spots were selected for further analysis. For each spot, MP scores were calculated using Seurat's ‘AddModuleScore’ function, and the spot was assigned to the MP with the highest score.

### 2.6 Calculation of the tumour neighbourhood score

The tumour neighbourhood score was developed to investigate the cell composition around individual tumour cell spots. As shown in Figure 3E, the neighbourhood was defined as the 11‐pixel area around the spot. The score for a cell type was calculated by summing the weights of that cell type within the neighbourhood.

To analyse the correlation between MPs and subcell types, subcell type scores were calculated with Seurat's ‘AddModuleScore’ function. The tumour neighbourhood score for a subcell type was found by summing scores around each tumour spot. The correlation between MP scores and subcell type neighbourhood scores was determined via Pearson's method.

### 2.7 Bulk RNA‐seq

The TCGA‐STAD dataset was retrieved from The Cancer Genome Atlas (TCGA) database (https://portal.gdc.cancer.gov). Metastatic transcriptome data from 66 paired primary and metastatic tumours were obtained from GSE237876. Transcriptional data on the immunotherapy response of 45 metastatic GC patients treated with anti‐PD‐1 therapy (PRJEB 25780 dataset) were obtained from the Tumor Immune Dysfunction and Exclusion database (http://tide.dfci.harvard.edu/).

For deconvoluting bulk sequencing data to estimate the cellular composition, the BayesPrism algorithm was applied, using previous GC scRNA‐seq data with 12 main cell types and 63 subcell types as references.^37^ The analysis excluded mitochondrial and ribosomal genes, with a focus on protein‐coding genes.

The epithelial expression matrix from BayesPrism was normalised with DESeq2 (v1.34.0) for further analysis, with MPs scores in each sample calculated by ssGSEA.

### 2.8 Pseudotime analysis

Monocle2 (v2.32.0) with the DDR‐Tree method was utilised to analyse single‐cell trajectories. The log‐normalised data were used as the input, and DEGs or marker genes of clusters were applied as ordering gene sets. Trajectories were deduced post‐dimension reduction and cell ordering. Additionally, the BEAM test was run to detect gene dynamics along branches.

### 2.9 Cell communication analysis

CellChat (v2.1.2) was used to analyse cell interactions in the TME. The communication probabilities were calculated using the trimean method via ‘computeCommunProb’, the cell–cell communications were filtered, and the pathway probabilities were computed with default parameters.

### 2.10 TME analysis

The cellular component abundances and microenvironment scores in the ST spot or bulk samples were deduced using the xCell pipeline (https://xcell.ucsf.edu/).

### 2.11 Survival analysis

To assess the impact of the abundance of each cell type on overall survival, we conducted Kaplan‐Meier survival analysis and log‐rank test statistics, utilising the survival (v3.5‐8) and survminer (v0.4.9) R packages. Cell type fractions and MP scores for the TCGA‐STAD cohort were determined using BayesPrism, as previously described. The ‘surv_cutpoint’ function from the survminer R package was utilised to determine the best cutpoint to divide each cell type into two groups.

### 2.12 Single‐cell drug susceptibility assessment

The Beyondcell (v2.2.1) R package was employed to ascertain drug sensitivities from scRNA‐seq data,^38^ leveraging the package's integrated drug perturbation signature collection database.

### 2.13 In vitro experiments

The methods of in vitro experiments are detailed in the Supporting Information.

### 2.14 Statistics and reproducibility

Statistical analyses of the data were conducted as described in the methods or figure legends. All the results from representative experiments were collected at least three times independently.

## 3 RESULTS

### 3.1 MPs within the GC malignant compartment

**FIGURE 1:**
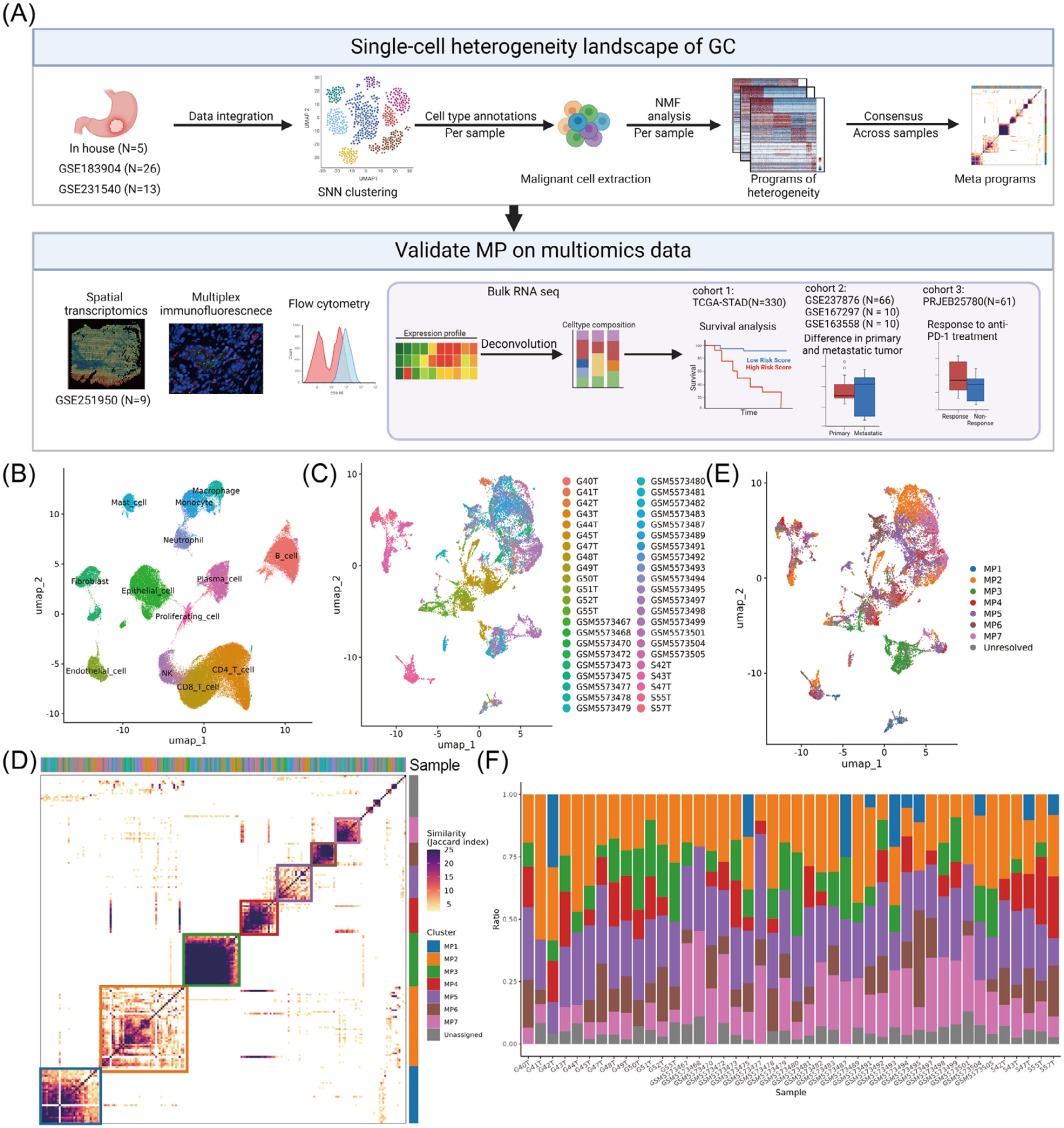
The single‐cell heterogeneity landscape of GC identifying seven MPs of tumour cells. (A) Scheme of the analysis workflow. (B) UMAP plot of 152 540 cells profiled by scRNA‐seq, coloured by cell type across 44 GC samples from three cohorts. (C) UMAP plot of 20 555 malignant cells, coloured by patient. (D) Hierarchical clustering analysis of pairwise similarities between NMF programs derived from malignant cells across all patients. The upper panel illustrates patient assignments, and the right panel delineates malignant meta programs identified from consensus NMF programs. Similarities between NMF programs were quantified by the Jaccard index over their signature genes. (E) UMAP plot of malignant cells, coloured by cell type identified according to the MP signature scores. (F) Bar plot showing the percentage distribution of each MP within individual patients.

To systematically reveal the tumour characteristics of GC tissue, we collected tumour tissue samples from five untreated GC patients and performed scRNA‐seq analysis of these samples by BD Rhapsody. To reduce the impact of patient heterogeneity and sequencing platforms, we incorporated the in‐house cohort with two public 10× Genomics scRNA‐seq cohorts (GSE183904 and GSE231540). Following the stringent quality control for each dataset and double‐cell removal, we ultimately obtained a total of 152 540 high‐quality single‐cell data from 44 GC samples (Figures 1A and S1 and Table S1). These cells were classified into 13 different cell types through dimensionality reduction and clustering (Figure 1B and Table S2).

Previous studies have reported significant heterogeneity in GC cells,^23^, ^39^, ^40^, ^41^ but their definition and function remain uncertain. We thus aimed to assess the diversity of the cellular state of malignant GC cells. Consistent with a previous study,^26^ 20 555 tumour cells were extracted for analysis and exhibited significant ITH according to their sample origin (Figure 1C). By performing NMF clustering on the malignant cells of each sample, seven unique meta patterns (MP1‐7), each containing a unique set of genes, were widely present in multiple samples reflecting common patterns of ITH (Figure 1D and Table S3). Based on gene sets of the MPs, we computed gene expression scores to define principal cellular states within malignant populations and quantify the proportional representation of each MP in individual patient samples. (Figure 1E,F).

**FIGURE 2:**
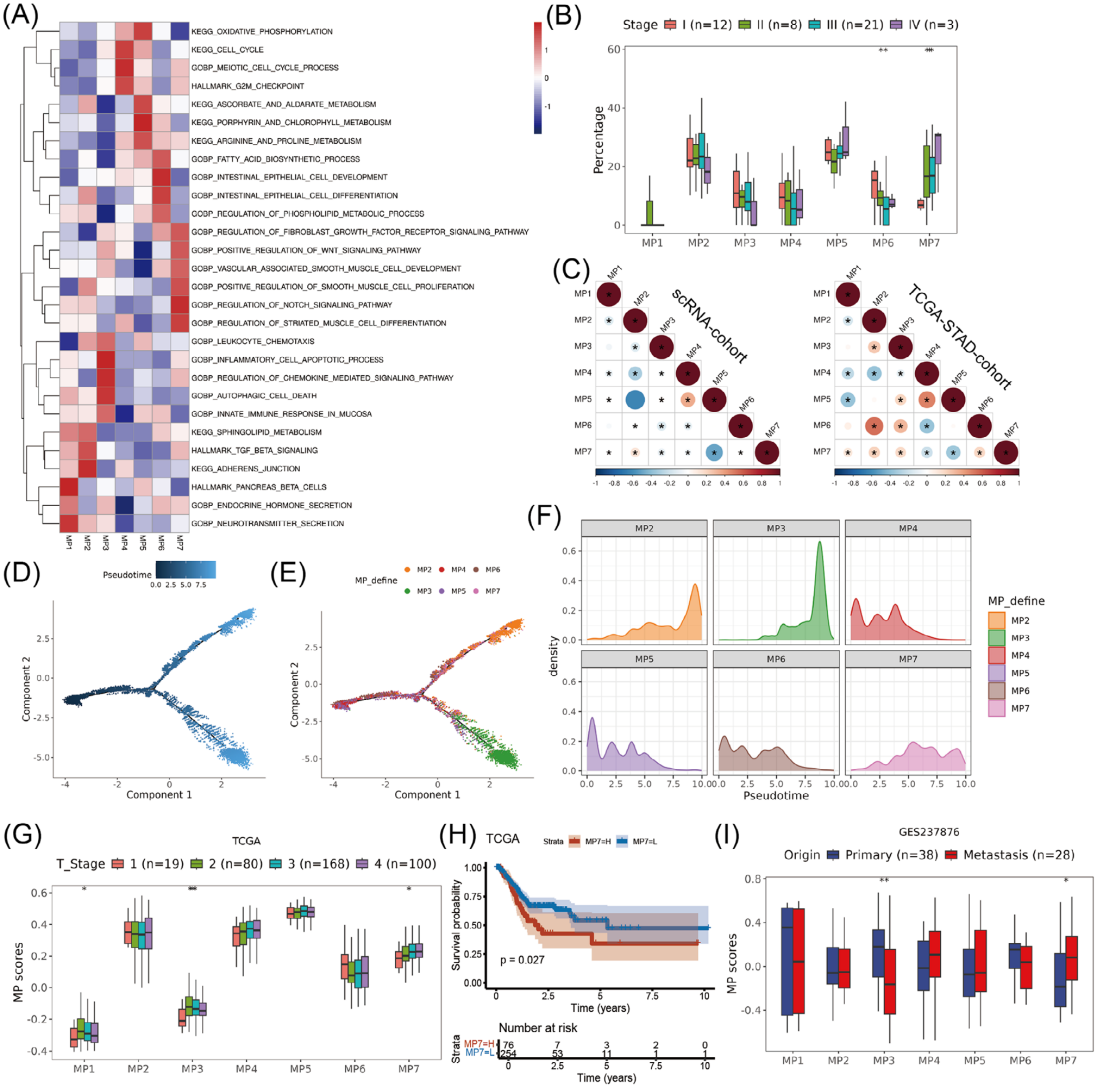
Characteristics of MPs and their correlations in GC. (A) Heatmap showing the represented GSVA scores of each MP in the enrichment of KEGG, GO and hallmark pathways. (B) Box plot showing the signature scores of MPs between different stages of GC. (C) Correlation heatmap displaying the Pearson correlation coefficients calculated among signature scores of distinct MPs in GC. The left heatmap shows the correlation of MP scores among tumour cells in single‐cell data. The right heatmap displays the correlation of MP scores across different samples in the TCGA‐STAD cohort. (D–F) Monocle pseudotime analysis of MPs. The distributions of the pseudotime order (D) and MP states (E) are shown and histogram panel showing MP distribution across pseudotime continuum (F). (G) Box plot showing the signature scores of MPs between different T stages of TNM in the TCGA‐STAD cohort. (H) Kaplan–Meier curves comparing the probability of survival according to the MP7 score in the TCGA‐STAD cohort. (I) Box plot showing the signature scores of MPs between primary and metastatic tissues in the GSE237876 cohort.

To elucidate the biological function and define the state of each MP, we performed functional enrichment analysis on malignant cells in different MPs (Figure 2A). MP1 was characterised by expression of genes (e.g., CHGA and CHGB) involved in the endocrine hormone secretion and neurotransmitter secretion pathways, thus representing classic neuroendocrine cells. MP2 harboured the distinctive genes CEACAM5, GKN1 and GKN2, which are indicative of intestinal metaplasia cells. MP3 was characterised by the expression of genes associated with the regulation of chemokine‐mediated signalling pathways and leukocyte chemotaxis (e.g., CCL5 and CXCR4), which might represent a subpopulation of epithelial cells involved in immune responses. MP4 had increased expression of genes associated with cell proliferation (e.g., TOP2A and MKI67). MP5 was enriched with ribosome related genes and characterised by active metabolic pathways, which probably reflected stress related cell state. MP6 displayed the hallmarks of mucous gland cells (e.g., MUC6, PGC and CXCL17) and was notably enriched in pathways related to intrinsic epithelial cell differentiation and the biosynthesis of fatty acids. Notably, MP7 exhibited a unique molecular signature enriched with histone‐related genes and key transcription factors (e.g., early growth response 1 [EGR1], SOX4 and ATF3), along with significant up‐regulation of tumour progression‐associated pathways, particularly positive regulation of Wnt and Notch signalling pathways,^42^, ^43^, ^44^ which were markedly enhanced in advanced GC (Figure 2B). Additionally, this subpopulation showed prominent activation of fibroblast differentiation and proliferation‐related pathways, including positive regulation of smooth muscle cell proliferation and fibroblast growth factor receptor signalling. These molecular signatures collectively indicate that MP7 likely represents a distinct tumour cell state with active crosstalk with CAFs.

Furthermore, to clarify the specificity and robustness of the seven MPs in GC, we conducted Jaccard similarity analysis with the pan‐cancer MPs. MPs in GC exhibited a significant level of congruence with pan‐cancer MPs, especially MP2, MP4, MP5 and MP7, indicating the stability of these GC MPs (Figure S2A). The MPs were subsequently validated in four seminal studies on GC tumour cell subpopulations (Figure S2B and Table S4). Consistent with previous studies, MP1 was recognised as a classic endocrine subtype, MP2 as a pit cell subtype, MP4 as a proliferation subtype and MP6 as a typical neck cell subtype. Notably, MP7 was associated with stress meta program (MP5) in pan‐cancer studies, sharing 14 common genes, including ATF3, EGR1, FOSB, GADD45B and NR4A1. In the context of GC, comparative analysis revealed that MP7 shared nine genes with the Proliferative Foveolar subpopulation identified by Cheng et al.,^41^ a non‐malignant cell state characterised by overlapping markers such as CXCL3, HIST1H2AG, SOX4 and NR4A1. However, MP7 demonstrated partial molecular similarity to the C3 tumour cell subpopulation described by Zhang et al.,^23^ sharing five key genes (KCNQ1OT1, GADD45B, FOSB, EGR1 and SOX4), an intermediate subgroup with poorly defined molecular features. Taken together, these results indicated that MP7 exhibited similarities to certain tumour cell subgroups, although the specific functions of these subgroups remain elusive.

Moreover, we utilised the R package Harmony to remove batch effects on malignant cells and re‐clustered malignant cells through a graph‐based clustering approach of the R package Seurat. We found that only the Cluster 2 and Cluster 4 subgroups in Seurat were exhibited a predominantly singular MP state (MP3 and MP6, respectively; Figure S2C–F). Collectively, these MPs were found to be both robust and adaptable, setting apart from previous single‐cell clustering studies on GC and illuminating the heterogeneity of GC cells from a novel perspective.

The correlations and progression paths of these GC MPs were further analysed. The MP7 exhibited negatively correlation with MP5, suggesting that these states were mutually exclusive (Figure 2C, the left correlation matrix). For pseudotime analysis, MP1 was excluded due to its high enrichment of genes specific to endocrine cells, and we found that MP4 and MP5 were at the beginning of the trajectory path, whereas MP2/MP7 cells and MP3 cells were at the end of the trajectory path (Figure 2D–F). Furthermore, we analysed the trajectories of these meta programs using branched expression analysis modelling (BEAM analysis) and hierarchical clustering to identify genes enriched across states (Figure S3A). Finally, we showed the characteristic changes of MP2 (CLDN18), MP3 (VIM), MP6 (REG1A) and MP7 (SOX4) marker genes over pseudo‐time (Figure S3B).

To gain a deeper understanding of the clinical implications of MP, we conducted an analysis of the MP scores within the TCGA‐STAD dataset. Consistent with previous single‐cell data, we observed that the MP7 score was significantly negatively correlated with the MP5 scores (Figure 2C, the right correlation matrix), while it was closely associated with tumour infiltration depth (Figure 2G) and poor prognosis in GC (Figures 2H and S3C). To further elucidate the correlation between MP7 and the invasion and metastasis of GC, we analysed the MP7 scores in GC tissues from various depths and found that the MP7 scores were greater in deep tumour tissues than that in superficial tissues (Figure S3D). Single‐cell data analysis of GC metastasis and primary tumours revealed an increased MP7 score in the metastatic sites (Figure S3E). Due to the limited number of scRNA‐seq samples, the correlation between MP7 and depth of invasion or metastatic foci did not reach statistical significance. To address this, we analysed bulk data from GC metastasis and primary tumour, which revealed significant up‐regulation of MP7 in metastases (Figure 2I). Collectively, these findings confirm the significant correlation between MP7 and GC progression, metastasis and poor prognosis, indicating that it represents the most malignant state of tumour cells.

### 3.2 Association of MPs with TME

**FIGURE 3:**
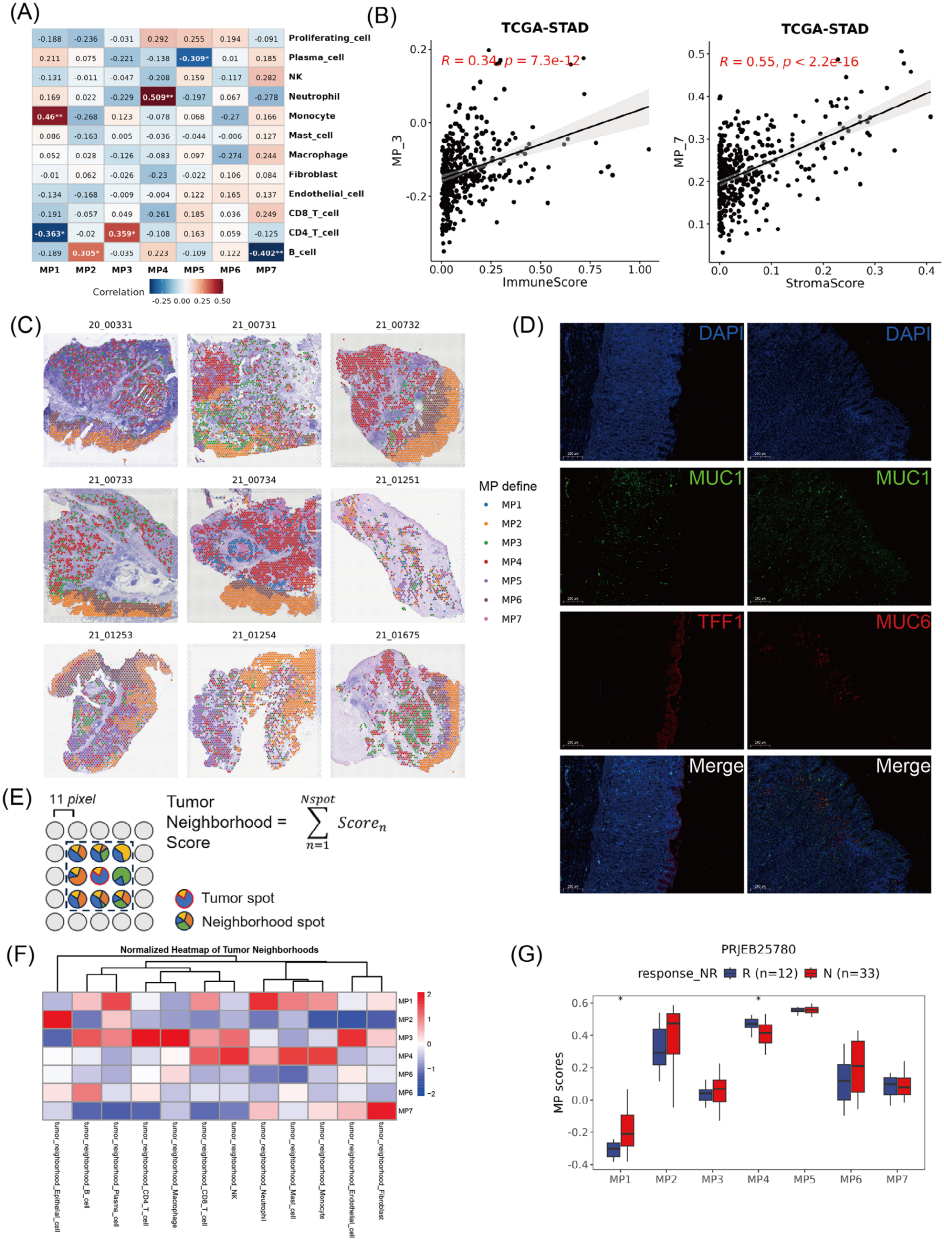
Correlations between MPs and different cell types in the TME. (A) Correlation heatmap displaying the Pearson correlation coefficients between each MP signature score and the fraction of each cell type in the TME. (B) Scatterplot displaying the Pearson correlation coefficients between the MP7 signature score and stroma score (left), and between the MP3 signature score and immunescore (right), as calculated via xCell in the TCGA‐STAD cohort. (C) The spatial distribution of epithelial meta programs, coloured by cell type identified according to MP signature scores. (D) Multiplexed immunofluorescence showing the localisation of MP2 (TFF1, red) and MP6 (MUC6, red). (E) Schematic plot and formula for calculating the tumour neighbourhood score. The red circle indicates the tumour spot of interest. The tumour neighbourhood is the eight spots (black sector) adjacent to the tumour spot of interest. (F) Heatmap showing Pearson's correlation coefficients between the signature scores of each MP and the neighbourhood scores of various TME cell types across 21 315 tumour spots within nine slides. (G) Boxplot showing each MP signature score between the responsive (R) and non‐responsive (N) groups of immunotherapies in the PRJEB25780 cohort.

The interaction between tumour cells and other cellular constituents within the TME is pivotal in the reconfiguration of the TME and the genesis of tumour heterogeneity.^45^ To further evaluate the relationship between the MPs and the TME, we correlated the summarised expression values of each MP with the percentages of TME cells inferred from the scRNA‐seq data within individual patients (Figure 3A). Interestingly, MP2 was positively associated with B cells, whereas MP7 displayed a negative correlation with B cells. MP3 was predominantly positively associated with CD4 T cells, while MP4 was positively correlated with neutrophil infiltration. Furthermore, we analysed the correlation between MP scores and TME score in the TCGA‐STAD data. The MP3 score was positively associated with immune scores, whereas the MP7 score was strongly correlated with the stromal score. (Figure 3B).

To gain a deeper understanding of the interaction between MPs and the TME at spatial resolution, we integrated our scRNA‐seq data with a ST data from nine GC samples using 10x Genomics Visium technology. After defined the MP state of each malignant spot (see section *Methods* and Figure 3C), MP2 was found to be predominantly localised to the superficial layer of the mucosa, showing distinct spatial aggregation, whereas MP6 was situated near MP2. MP3, MP4, MP5 and MP7 presented in the submucosal layer of tumours and exhibited diffuse distribution, which facilitated their spatial engagement with diverse cell types in the TME. Furthermore, we have performed analyses focusing specifically on the tumour–stromal interface (Figure S4A) and found that MP2 was predominantly localised within the tumour region, while MP3 and MP7 show enrichment at the tumour–stromal interface (Figure S4B). Additionally, we examined the expression of marker genes that distinguish MP2 and MP6 by mIF. Tumour cells with high TFF1 expression (MP2 state) were clustered mainly at the top of the mucosal layer, while MUC6‐positive cells (MP6 state) were predominantly aggregated around the neck of the gland lumen (Figure 3D).

To study the composition of TME cells associated with each MP, we defined a cell neighbourhood score by calculating the cell‐type score surrounding the corresponding spot^27^ (Figure 3E). In all nine ST slides, MP3 spots presented elevated immune cell neighbourhood scores, such as those for CD4 T cells, CD8 T cells, NK cells and macrophages (Figure 3F). Surprisingly, cytotoxic immune cells, especially CD8 T cells and NK cells, were mainly aggregated around MP4 cells, confirming that the MP3 and MP4 spots may be involved in different tumour immune responses. Moreover, MP7 spots were surrounded primarily by accumulated fibroblasts, indicating their key role in matrix remodelling. Concurrently, there was a significant decrease in the enrichment of immune cells, including B cells, CD4 T cells, CD8 T cells and NK cells, which suggested that fibroblasts surrounding MP7 might create an immune‐deprived microenvironment around it (Figure 3F). Additionally, we analysed tumour neighbourhood scores at the tumour–stromal interface versus whole‐tumour analysis. As shown in the Figure S4C, the interface scores closely matched the whole‐tumour results: MP2 maintained epithelial cells and plasma cells; MP3 enriched CD4 T cells, endothelial cells and macrophages; MP4 exhibiting elevated CD8 T cells, mast cells, monocytes and NK cells; and MP7 accumulated fibroblast. These results confirm the consistency of the whole‐tumour findings, strengthening the reliability of our conclusions.

Based on these observations above, we subsequently investigated the potential roles of MPs and TME components in immunotherapy. By analysing the groups responsive and non‐responsive to PD‐1 immunotherapy in the PRJEB25780 dataset, we found that fibroblasts were significantly enriched in the group non‐responsive to PD‐1 treatment (Figure S4D). MP4, which has the closest spatial distance to immune killer cells such as CD8 T cells and NK cells, significantly increased in the responsive group, whereas there was no significant difference in the scores of MP3 and MP7 between the two groups (Figure 3G). Together, these findings suggest that MP3 is closely associated with tumour immune infiltration, MP4 more effectively reflects the cytotoxic capacity of immune cells compared with MP3, and MP7 is characteristically related to fibroblast infiltration.

### 3.3 MP3 and MP4 were involved distinct functional states of CD8 T cells

**FIGURE 4:**
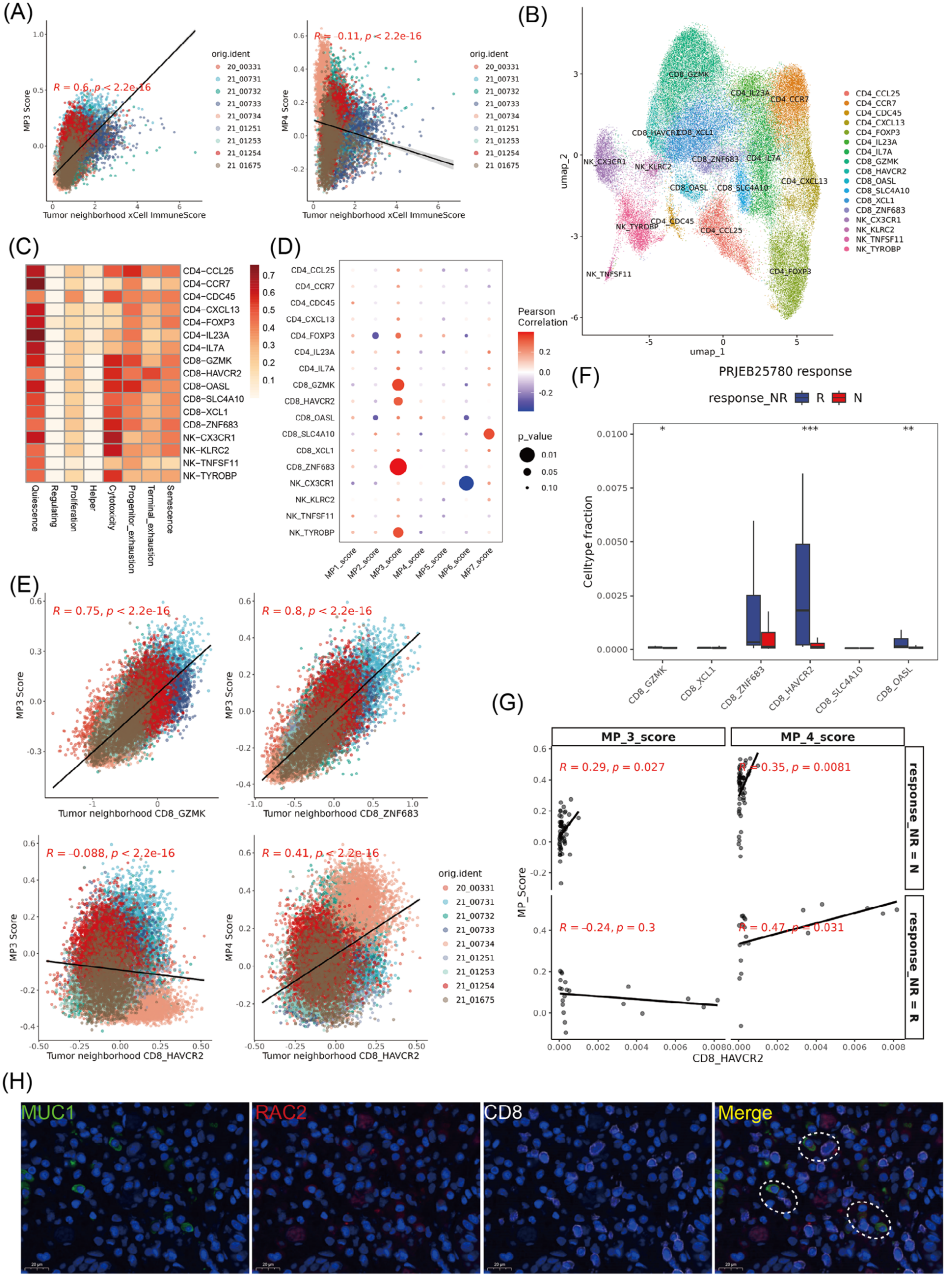
MP3 and MP4 are associated with distinct CD8 T cells skewed toward a cytotoxic or exhaustion state. (A) Scatterplot displaying the Pearson correlation coefficients between the MP3 and MP4 signature score and the tumour neighbourhood immunescore. (B) UMAP plot of T and NK cells. (C) Heatmap showing the functional scores of each T cell and NK cell clusters calculated by TCellSI. (D) Correlation heatmap displaying the Pearson correlation coefficients between each MP signature score and fractions of T and NK cells in the TME. (E) Scatter plots showing Pearson's correlation between the gene signature scores of MP3 or MP4 and the scores of cytotoxic or exhausted CD8 T cells neighbouring the tumour spots across nine slides. (F) Boxplot showing each CD8 T cluster between the responsive (R) and non‐responsive (NR) groups in the PRJEB25780 cohort. (G) Scatter plots showing Pearson's correlation between the M3 or MP4 score and exhausted T cells (CD8‐HAVCR2) across different groups with varying responses to immunotherapy. (H) Multiplexed immunofluorescence images showing the interaction between MP3 (RAC2) and CD8 T cells (CD8, white). The dashed circles indicate areas where CD8 T cell and MP3 tumour cells are spatially adjacent.

In ST, significant enrichment of T cells and NK cells was observed surrounding both MP3 and MP4 (Figure 3F). However, MP3 scores were positively correlated with immune scores, while MP4 scores demonstrated a negative correlation, suggesting distinct immune response mechanisms mediated by these cell populations (Figure 4A). To clarify the distinct immune activities as characteristic responses of the two states of tumour cells, we performed further dimensionality reduction and clustering on NK and T cells, revealing seven subtypes of CD4 T cells, six subtypes of CD8 T cells and four subtypes of NK cells (Figure 4B). Furthermore, all subpopulation states were evaluated via TCellSI^46^ (Figure 4C), and the correlations between the MP scores and the proportions of immune cell subgroups in the single‐cell data were compared (Figure 4D). MP3 exhibited a strong correlation with diverse CD8 T cell populations, particularly the cytotoxic subgroups, such as CD8‐GZMK, CD8‐HAVCR2 and CD8‐ZNF683. In the spatial transcriptome samples, by evaluating the expression of CD8 T cell signatures in MP3 or MP4 neighbourhoods, we observed a significant positive correlation between MP3 and CD8‐GZMK or CD8‐ZNF683, which was consistent with previous results (Figure 4E, upper). However, the increase in the tumour neighbourhood CD8‐HAVR2 score was not related to the MP3 score but rather to the MP4 score (Figure 4E, bottom), indicating that MP3 and MP4 seem to be associated with different functional states of CD8 T cells. Moreover, within the GC immunotherapy dataset, we observed significant enrichment of exhausted T cells (CD8‐HAVCR2) and MP4 scores in the anti‐PD1 treatment response cohort, whereas MP3 showed no significant difference between responders and non‐responders to immunotherapy (Figures 4F and 3G). Furthermore, MP4 and CD8‐HAVCR2 displayed the highest correlation in the immune‐responsive group, outperforming their correlation in the non‐responsive group, as well as MP3 and CD8‐HAVCR2 in both groups (Figure 4G). These findings indicate that although MP3 shows a significant correlation with immune cell infiltration in GC, MP4 appears to be more strongly associated with immune elimination function of CD8 T cells.

Additionally, to highlight the differences between the MP3 and pan‐cancer immune programs, we analysed their spatial expression patterns. MP3 predominantly displayed features characteristic of the tumour epithelium (Figure S4A,B). This contrasted to the immune score and CD8 T cell signature, indicating that MP3 tends to be more prevalent at the tumour periphery. Using mIF in GC tissue (Figure 4H), we observed frequent co‐localisation of CD8 T cells with MP3 tumour cells (dashed regions). Moreover, TIP analysis of MP3‐dominant tumours revealed CD8 T cell infiltration in tumours with high RAC2 expression (Figure S5C). Flow cytometry of isolated epithelial cells further confirmed MP3 presence in GC tissue, with characteristic markers (RAC2, CXCR4 and IL‐7R) detected in a subset of epithelial cells (Figure S5D). Together, these findings indicate that MP3 defines an epithelial subpopulation engaged in immune interactions, ruling out technical artifacts or immune cell contamination.

### 3.4 MP7 spots harboured an immune lockdown microenvironment and associated with myCAFs

**FIGURE 5:**
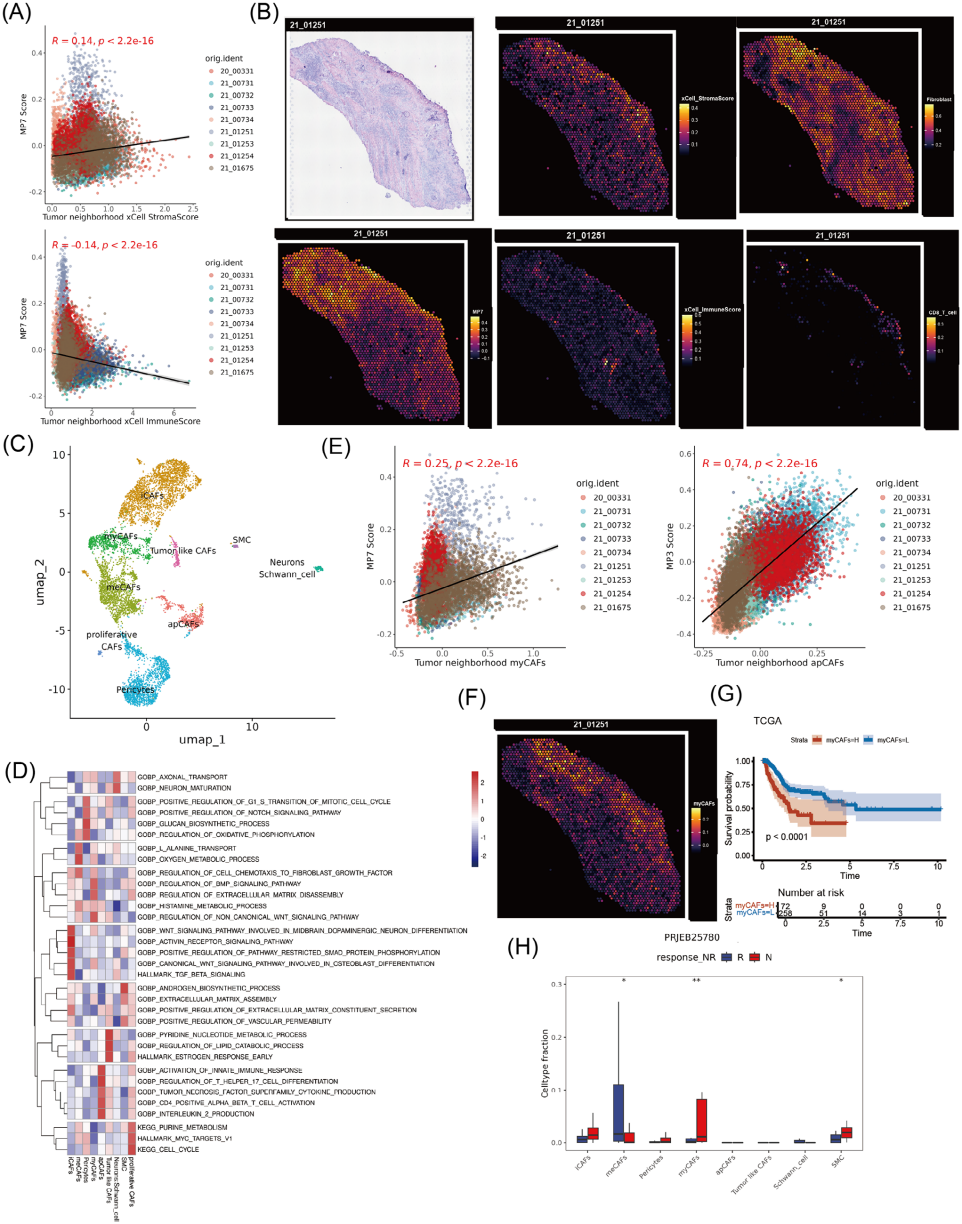
MP7 spots harboured an immune lockdown microenvironment and associated with myCAFs. (A) Scatter plots showing Pearson's correlation between the MP7 score and the tumour neighbourhood immunescore or stromascore. (B) Spatial distributions of the MP7 score, immunescore, stromascore, fibroblast and CD8 T cell inflamed gene expression profiles in representative slide 21_01251. (C) UMAP plot of fibroblasts, coloured by cell subtype. (D) Heatmap showing the represented GSVA scores of each subtype in the enrichment of KEGG, GO and hallmark pathways. (E) Scatter plots showing the Pearson's correlation between the MP7 score and the tumour neighbourhood myCAFs or the Pearson's correlation between the gene signature score of MP3 and the score of the tumour neighbourhood apCAFs across nine slides. (F) Spatial distribution of myCAFs in representative slide 21_01251. (G) Kaplan–Meier curves comparing the probability of survival according to myCAFs score in the TCGA‐STAD cohort. (H) Boxplot showing each fibroblast subtype between the responsive (R) and non‐responsive (N) groups of immunotherapies in the PRJEB25780 cohort.

The aforementioned findings indicated that fibroblasts predominantly accumulated around MP7, while NK and T cells were negatively correlated with MP7 (Figure 3F). We further analysed both the stromal score and the immune score around MP7 and found that the stromal score around MP7 significantly increased; conversely, the immune score significantly decreased, indicating the formation of an immuno‐deprived microenvironment around MP7 (Figure 5A). Additionally, we characterised the expression of marker genes that distinguish various MPs and TIPs. Notably, EGR1‐high tumours (MP7‐dominant) exhibited strong enrichment of the CAF marker FAP and a striking absence of CD8 T cell infiltration. In contrast, RAC2‐high tumours (MP3‐dominant) showed lacked FAP expression and maintained abundant CD8 T cell infiltration (Figure S5D). We next presented a ST sample enriched with MP7 and observed a significant decrease in immune scores; in particularly, CD8 T cells were present only at the tumour margins, indicating a significant disruption in the infiltration process, which may be caused by the significant enrichment of fibroblasts (Figure 5B).

To strengthen the evidence that CAFs promoted the formation of an immunologically isolated microenvironment around MP7, we proceeded to perform further dimensionality reduction and clustering on fibroblast subsets. We identified a group of Schwann cells and eight clusters of fibroblasts, including inflammatory CAFs (iCAFs, Fib_C01), metabolic CAFs (meCAFs, Fib_C02), myofibroblasts (myCAFs, Fib_C04), antigen‐presenting CAFs (apCAFs, Fib_C05), tumour‐like CAFs (Fib_C06), proliferative CAFs (Fib_C09), pericyte (Fib_C03) and smooth muscle cells (SMC, Fib_C08) (Figure 5C,D). By analysing the relationship between MP scores and CAF subgroup proportions, we found that MP7 scores escalated with increasing proportions of iCAFs; in contrast, MP4 scores exhibited a markedly negative correlation with iCAFs (Figure S6A). Spatial distribution analysis revealed that iCAFs predominantly aggregated around MP2 and MP5, whereas the scores for iCAFs around MP7 notably decreased (Figure S6B,C). The discrepancy between ST and single‐cell transcriptomic data may arise from limitations in sample size or analytical methodologies, but it also likely reflects intrinsic, tumour state‐dependent preferences in CAF spatial distribution within the TME. Additionally, apCAFs were more dominant around MP3, potentially participating in tumour immune responses (Figures 5E and S6B). Notably, myCAFs were predominantly enriched around MP7 (Figures 5E and S6B), and the ST image revealed that myCAFs were primarily situated between the tumour and CD8 T cells, constituting the main subtype of CAFs that undergo immune exclusion (Figure 5F).

To elucidate the contribution of different CAF subgroups to tumour progression, the prognostic implications of each fibroblast subgroup within the TCGA‐STAD dataset was examined. Elevated levels of iCAFs were associated with a favourable prognosis, while a higher prevalence of myCAFs was linked to a poor outcome (Figures 5G and S6D,E). Additionally, in the GC immunotherapy cohort, there was a marked increase in the prevalence of myCAFs among patients who did not respond to PD‐1 treatment, suggesting that myCAFs might contribute to the resistance to immunotherapy through the promotion of immune exclusion (Figure 5H). Consequently, myCAFs, the predominant CAF subtype surrounding MP7, likely contribute to immune evasion mechanisms, particularly in the microenvironment adjacent to MP7‐type GC cells.

### 3.5 MP7 cells recruited fibroblasts and induced them to myCAFs via GDF15

**FIGURE 6:**
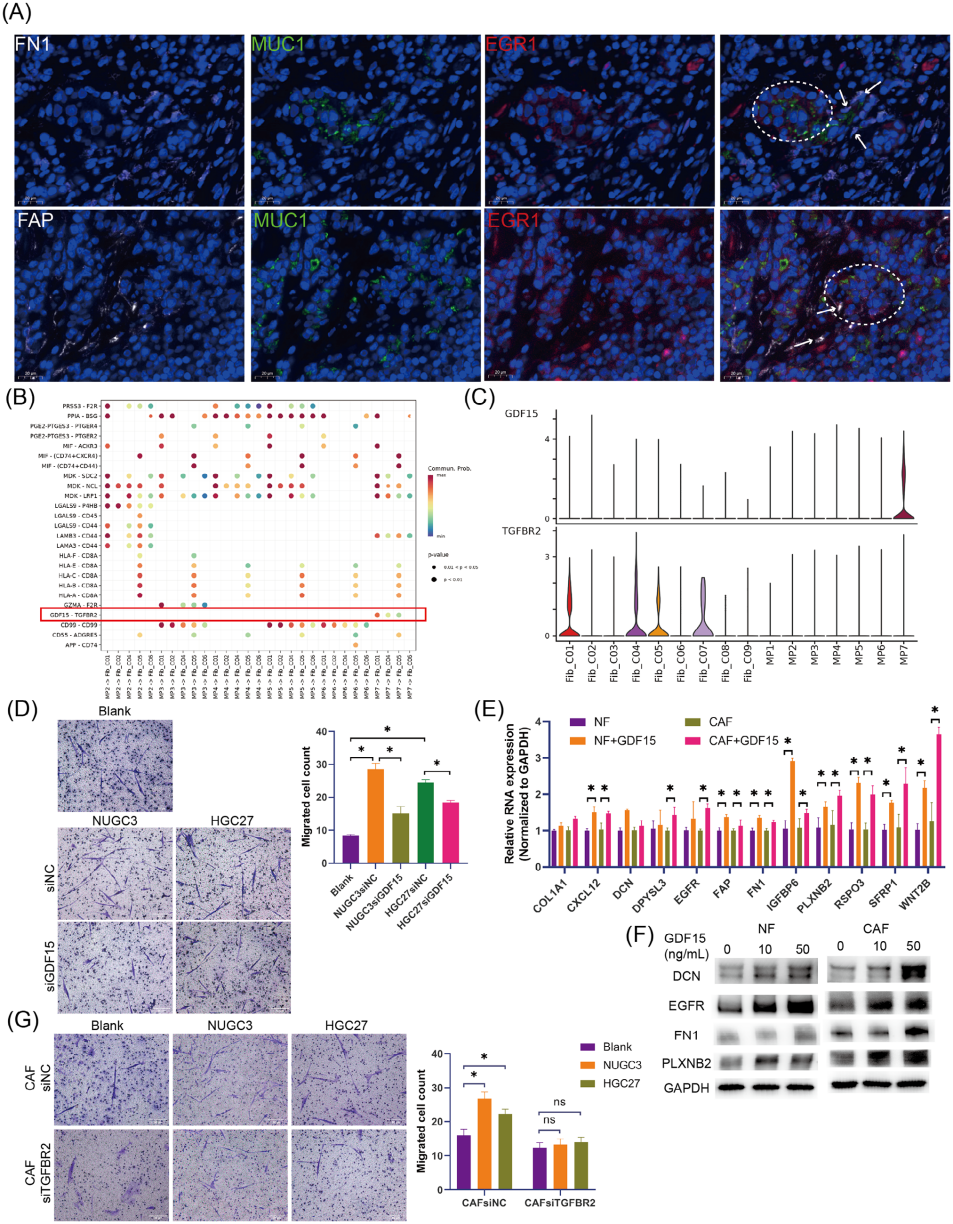
GDF15 contributes to the generation of myCAFs surrounding MP7. (A) Multiplexed immunofluorescence images showing the interaction between MP7 (EGR1) and myCAFs (FN1 or FAP). The dashed circle delineates regions of MP7 tumour cell aggregation, and the arrows indicate myCAFs in close to MP7 cells. (B) Dot plot showing selected interaction pairs between MPs and fibroblast subtypes. (C) Violin plots displaying the expression levels of GDF15–TGFBR2 ligands in subpopulations of tumour cells and fibroblasts. (D) Transwell assay showing the chemotactic effect of the conditioned medium from GDF15‐knockdown GC cells (siGDF15‐1) on CAFs. (E and F) PCR (E) and Western blotting (F) assays showing the promotional effect of exogenous GDF15 on the expression of myCAFs markers in NFs and CAFs. (G) Migration assays demonstrating the effect of GC conditioned medium on the migratory activity of NFs and CAFs after TGFBR2 knockdown (siTGFBR2‐1). Data from three independent experiments are presented as bar graphs showing with the mean ± SEM.

In the mIF images of GC, myCAFs (Figure 6A, arrows, FN1^+^ cells or FAP^+^ cells) were confirmed to be significantly enriched around the MP7 subpopulation (Figure 6A, dashed circle, MUC1^+^ EGR1^+^ cells). Further exploration of the potential molecular mechanisms involved in the aggregation of myCAFs around MP7 was conducted using CellChat. We observed that GDF15 derived from MP7 significantly interacted with the TGFBR2 receptor on the surface of CAF subpopulations, including iCAFs (Fib_C01), myCAFs (Fib_C04) and apCAFs (Fib_C04), exhibiting distinct MP subpopulation specificity (Figure 6B,C). To determine whether GDF15 is involved in the aggregation of myCAFs around MP7, we first used the supernatant of GC cells or exogenous GDF15 to induce chemotaxis in GC fibroblasts. Transwell assay revealed that both the supernatants from GC cell lines (NUGC3 and HGC27) and exogenous GDF15 could recruit CAFs and normal fibroblasts (NFs) (Figure S7A). Furthermore, we employed siRNA to knockdown of GDF15 expression in GC cells (Figure S7B) and assessed the chemotactic effect of the tumour cell supernatant on CAFs. We observed that the supernatant from NUGC3 and HGC27 cells with GDF15 knockdown exhibited a significant reduction in their ability to induce CAF migration (Figure 6D). These findings confirm that the induction of CAFs by GC cells is dependent on GDF15. Besides, GDF15 significantly promoted the expression of DCN (decorin), EGFR (epidermal growth factor receptor), FN1 (fibronectin 1) and PLXNB2 (plexin B2) in both NFs and CAFs, indicating that GDF15 could induce the switch to myCAFs (Figure 6E,F).

Subsequently, we investigated the primary receptor mediating GDF15‐induced CAF chemotaxis. Expression analysis revealed that of TGFBR2, identified by CellChat as the primary GDF15 receptor, was significant up‐regulated in both GC tissues and CAFs, while GFRAL, another known GDF15 receptor, was nearly undetectable in GC tissues and CAFs (Figure S7C,D). These findings established TGFBR2 as the predominant receptor for GDF15 in GC. To further elucidate the role of TGFBR2 in GDF15‐mediated chemotaxis, we knocked down TGFBR2 in CAFs. While TGFBR2 depletion did not significantly altered the intrinsic migratory ability of CAFs, it markedly attenuated GDF15‐induced chemotaxis (Figure S7E,F). Consistent with this observation, the chemotactic effect of NUGC3 and HGC27 cell supernatants on CAFs was significantly reduced following TGFBR2 knockdown (Figure 6G). Thus, these findings suggest that MP7 induces the aggregation of NFs and CAFs and then promotes their transition to myCAFs through the GDF15–TGFBR2 ligand–receptor interaction.

### 3.6 MyCAFs‐derived RSPO3 facilitated the formation of the MP7 state via EGR1

**FIGURE 7:**
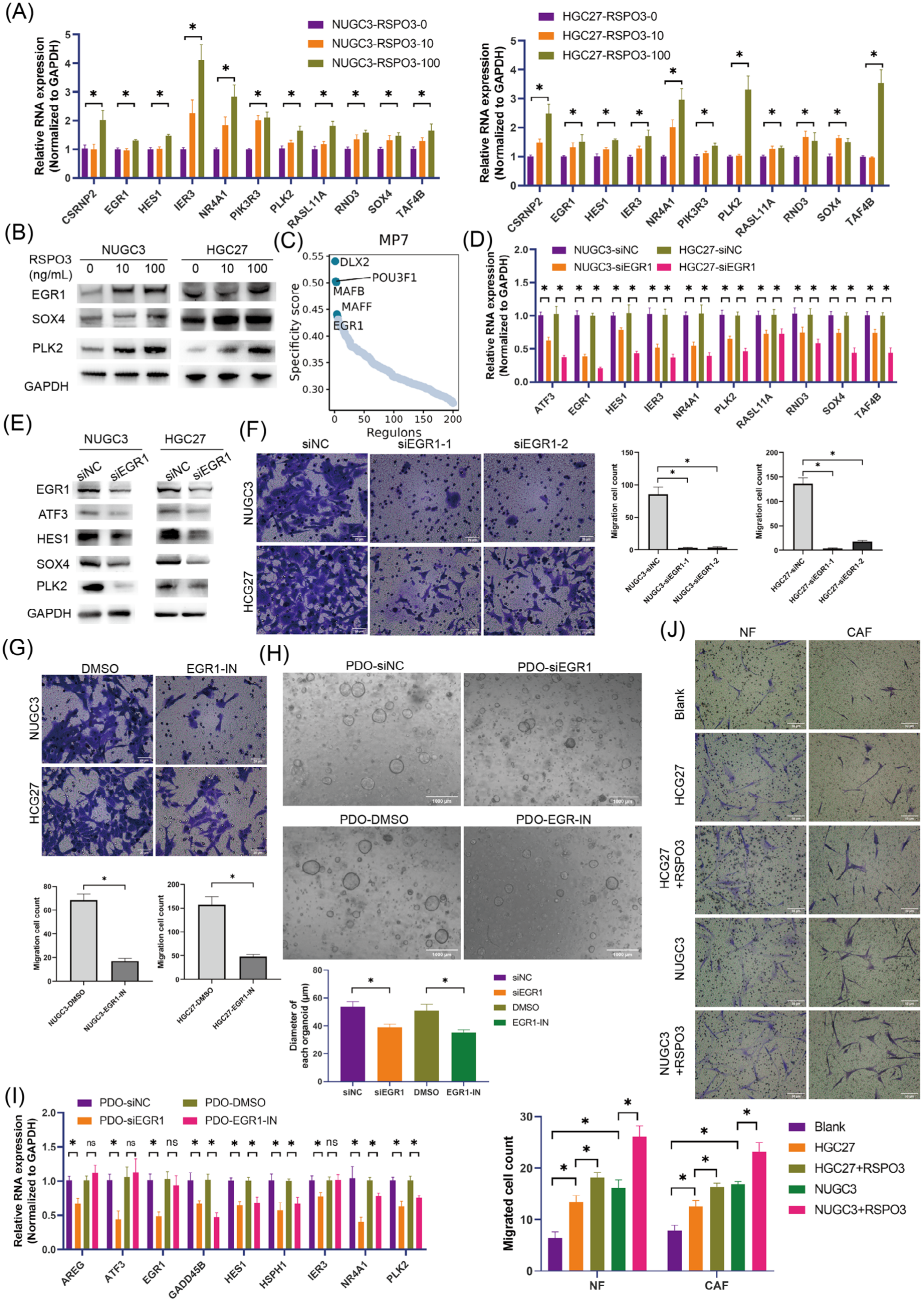
EGR1 is critical for MP7 transformation and regulated by RSPO3 derived from myCAFs. (A) Quantification of MP7 genes by qPCR in GC cells after exposure to exogenous RSPO3. (B) Western blot of the MP7 protein in GC cells after exposure to exogenous RSPO3. (C) Scatter plot showing the top five specific transcriptional regulons in MP7. (D) Bar plot displaying the MP7 gene in GC cells after EGR1 knockdown via EGR1 siRNA (siEGR1‐1) by qPCR. (E) Western blot of the MP7 protein in GC cells after EGR1 knockdown by EGR1 siRNA (siEGR1‐1). (F) Transwell experiment showing the migration of GC cells after EGR1 knockdown. (G) Transwell analysis showing the migration of GC cells after treatment with an EGR inhibitor (EGR1‐IN). (H) Morphological photography of GC PDOs after EGR1 depletion by siRNA (siEGR1‐1) or treatment with EGR1‐IN for 10 days and the size of PDOs was quantified by analysing their diameters. (I) Quantification of MP7 genes by qPCR in GC PDOs after EGR1 depletion by siRNA (siEGR1‐1) or treatment with EGR1‐IN for 10 days. (J) Transwell assay showing the effects of RSPO3‐pretreated HGC27 or NUGC3 cells on the migration ability of NFs and CAFs. Data from three independent experiments are presented as bar graphs showing with the mean ± SEM.

To determine the potential involvement of myCAFs in the development of GC cells, pathway enrichment analysis was conducted in myCAFs. ECM receptor interaction, Wnt signalling pathway and TGFβ signalling pathway were significantly enriched in myCAFs (Figure S7G,H). Notably, RSPO3 (R‐spondin‐3), a potent activator of the canonical Wnt pathway, was markedly increased in myCAFs induced by GDF15 (Figure 6E), indicating its potential as a key regulator in myCAFs‐mediated modulation of GC cells. Consequently, the impact of exogenous RSPO3 on GC cells was assessed. The results demonstrated that RSPO3 markedly enhanced the expression of MP7 markers in the GC cell lines NUGC3 and HGC27 (Figure 7A,B).

Furthermore, to elucidate the key TF of MP7 transformation, we performed an enrichment analysis of TF activity in MP7 cells and identified EGR1 as a highly active TF, as well as a marker gene of MP7, potentially playing a crucial role in the transformation or maintenance of the MP7 cell state (Figure 7C). We then silenced EGR1 in the GC cell lines NUGC3 and HGC27 via siRNA and observed a significant decrease in the expression of MP7 marker genes, such as ATF3, HES1, NR4A1 and SOX4 (Figure 7D,E). Moreover, EGR1 knockdown significantly inhibited the migration ability of GC cells (Figure 7F). Consistent with these findings, treatment with EGR1 inhibitor (EGR1‐IN) also resulted in a markedly reduction of GC cell migration (Figure 7G). In GC patient‐derived organoids (PDOs), EGR1 knockdown and pharmacological inhibition of EGR1 were also led to a significant reduction in the size of PDOs, along with a decrease in the expression of marker genes for MP7 (Figure 7H,I), indicating the crucial role of EGR1 in the formation and maintenance of MP7. Additionally, the chemotactic effect of GC cells pretreated with RSPO3 (MP7) on CAFs was assessed. The results showed that the migratory capacity of CAFs and NFs induced by RSPO3‐pretreated HGC27 and NUGC3 cells was enhanced (Figure 7J). The above results suggest that myCAFs‐derived RSPO3 facilitated the formation of MP7 state via EGR1.

### 3.7 MP7 served as a potential therapeutic target in GC treatment

**FIGURE 8:**
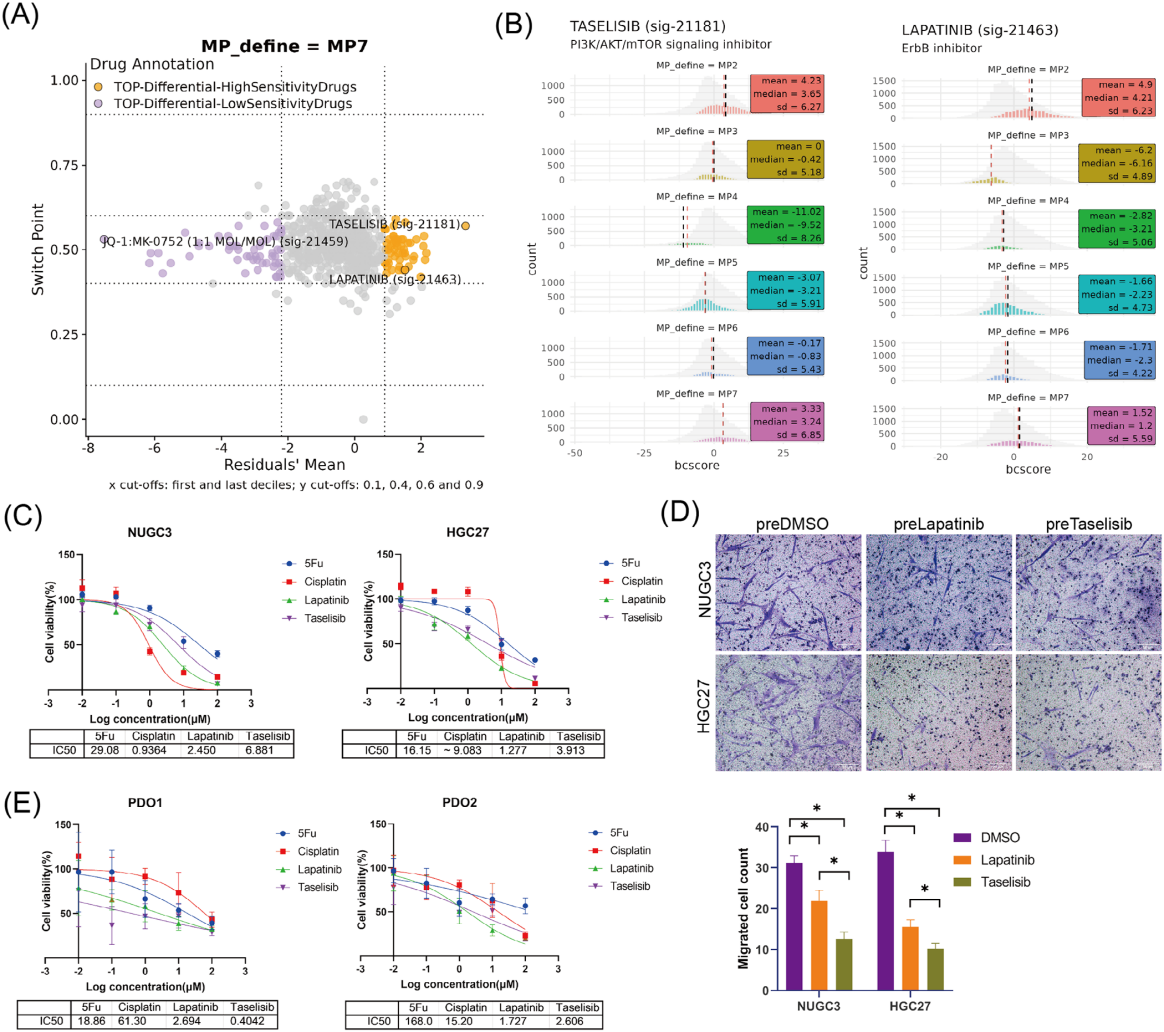
Identification of candidate drugs for MP7. (A) Four squares plot showing the drugs to which MP7 cells are differentially insensitive or sensitive, as estimated by Beyondcell. (B) Histogram representing taselisib and lapatinib Beyondcell scores in each MP. (C) IC50 values for 5‐Fu, cisplatin, taselisib and lapatinib in cytotoxicity assays with GC cells. (D) Transwell analysis showing the CAF chemotaxis migration stimulated by equal numbers of taselisib and lapatinib pre‐treated GC cells. (E) IC50 values for 5‐Fu, cisplatin, taselisib and lapatinib in cytotoxicity assays with GC PDO.

MP7 tumour cells, characterised by their high malignancy and propensity to induce myCAFs transformation, represented promising therapeutic targets for GC. While EGR1‐IN was initially considered as a candidate compound for targeting MP7, in vitro experiments revealed no significant cytotoxic effect on GC cells (Figure S8A). To find more effective drugs targeting MP7, we used BeyondCell^38^ to analyse drug sensitivity profiles. Tumour cells were categorised into three different BC clusters based on drug sensitivity (Figure S8B). Notably, MP3 displayed unique drug sensitivity, while MP2 and MP7 exhibited similar sensitivity (Figure S8C). For the MP7 cell subpopulation, taselisib, a potent and selective inhibitor of phosphoinositide 3‐kinase (PI3K),^47^, ^48^ and lapatinib, a dual tyrosine kinase inhibitor that inhibits both EGFR and HER2,^49^, ^50^ were predicted as highly sensitive drugs (Figure 8A,B).

To elucidate the relationship between MP7 transformation and PI3K/AKT signalling pathway activation, we first attenuated the MP7 state by depleting EGR1 in GC cells. EGR1 down‐regulation significantly reduced the phosphorylation of PI3K, AKT, ERK and MAPK, indicating that EGR1 regulates MP7 transformation through activation of the PI3K/AKT signalling pathway (Figure S8D).

We further assessed the cytotoxic effect of taselisib and lapatinib on MP7 using two conventional drugs of GC, cisplatin and 5‐Fu, as controls. In NUGC3 cells, the IC50 values for lapatinib and taselisib were lower than that of 5‐Fu, while in the HGC27 cells, both drugs exhibited lower IC50 values compared with 5‐Fu and cisplatin, demonstrating their potent cytotoxic effects against GC cells (Figure 8C).

To explore whether these drugs impair MP7‐mediated CAF recruitment, NUGC3 and HGC27 GC cell lines were pretreated with IC50 concentrations of lapatinib and taselisib for 48 h. CAF chemotaxis assays revealed a significant reduction in CAF recruitment by drug‐pretreated tumour cells compared with the DMSO‐pretreated control group (Figure 8D). Meanwhile, taselisib‐pretreated GC cells exhibited a more pronounced attenuation of CAF chemotactic induction than lapatinib‐pretreated cells, likely due to the stronger targeting effect of taselisib on MP7 cells (Figure 8D).

Additionally, both drugs were tested in GC PDOs. In PDOs derived from two patients, both lapatinib and taselisib demonstrated higher sensitivity compared with 5‐Fu and cisplatin, suggesting their potential therapeutic value in GC treatment (Figure 8E). Collectively, these findings highlight MP7 as a potential therapeutic target in GC and suggest that taselisib, with its high specificity and efficacy, may provide a promising strategy for MP7‐targeted GC therapy.

## 4 DISCUSSION

GC is recognised for its pronounced heterogeneity, which influences tumour progression and response to clinical therapies.^51^, ^52^ However, its underlying molecular mechanisms remain poorly understood. In this study, we utilised the MP approach to delineate ITH in GC and integrated ST to elucidate the biological significance of each MP state. Among the seven cellular states we identified, MP3 and MP4 were intimately associated with the tumour microenvironment, particularly in their spatial co‐localisation with CD8 T cells, which classifies them as immune‐responsive cellular states. MP7 was significantly increased in advanced GC and metastasis tissue, and the aggregation of myCAFs around MP7 shielded it from immune elimination and endowed it with a high metastatic propensity. The intimate spatial association and interplay between the metastatic program MP7 and myCAFs mediated by GDF15/RSPO3 led to fewer infiltration of CD8 T cells and created an immune‐deprived microenvironment in GC. Targeting MP7 tumour cells by taselisib and lapatinib or their interaction loop with myCAFs may provide a basis for clinical decision‐making and development of novel anti‐tumour strategies.

Our research revealed that while CD8 T cells were enriched around both immune‐related MP3 cells (positive for RAC2, CCL5 and CXCR4) and proliferation‐related MP4 cells (positive for TOP2A and MKI67), it is noteworthy that MP3 cells did not show a significant increase in the group responding to PD‐1 therapy, suggesting that MP3 may possess unique mechanisms for immune evasion that are not yet understood. Studies have shown that CCL5, a marker of MP3 shown in the present study, may contribute to the accumulation of CD8 T cells^53^, ^54^; however, elevated CCL5 levels are linked to an unfavourable prognosis and hinder the ability of CD8 T cells to eradicate tumours in GC.^55^, ^56^, ^57^, ^58^ Furthermore, CXCR4, another marker gene of MP3, has also been confirmed to be closely related to poor prognosis in GC,^59^ and CD133^+^CXCR4^+^ lung cancer cells show higher metastatic and chemo‐resistant properties.^60^, ^61^ In pancreatic cancer, inhibiting CXCR4 has been shown to enhance T cell infiltration into tumours and synergise with anti‐PD‐1 therapies.^62^ These findings suggest that while MP3 is associated with immune cell infiltration, it does not appear to be a primary target for CD8 T cell‐mediated tumour elimination. However, its unique characteristics may hold potential value in enhancing the effectiveness of immunotherapy for GC.

The MP7 state was markedly elevated in metastases and strongly correlated with poor prognosis of GC. Our study identified EGR1 as a pivotal transcription factor in the regulation of MP7 formation. EGR1 is instrumental in cellular development, growth, proliferation and fibrosis and is triggered by various extracellular signalling molecules, including growth factors and cytokines.^63^ Within tumours, EGR1 promotes metastasis and drug resistance by stimulating the generation of tumour stem cells.^17^, ^64^, ^65^ We found that EGR1 enhances the expression of MP7 markers such as SOX4, NR4A1, ATF3 and HES1, promoting the metastatic potential of GC cells. To counteract this transformation, EGR1 inhibitors were employed to prevent the emergence of MP7. Our experiments demonstrated that EGR1‐IN can significantly suppress the formation, maintenance and metastatic capabilities of MP7 GC cells. Moreover, in PDOs, we observed that knockdown or inhibition of EGR1 led to a significant reduction in organoid size, accompanied by a marked decrease in MP7 marker expression. Additionally, taselisib, a potent and selective inhibitor of PI3K, and lapatinib, a dual tyrosine kinase inhibitor targeting EGFR1 and HER2, have emerged as promising therapeutic agents by targeting MP7. Previous studies have shown that lapatinib combined with paclitaxel significantly increases the overall response rate in patients with HER2‐positive GC, and in patients with HER2 FISH‐positive IHC 3+, the risk of disease progression and death is markedly lower with combination therapy than with paclitaxel alone.^66^ Moreover, the addition of lapatinib to the CapeOx (capecitabine and oxaliplatin) regimen, prolonged overall survival, particularly in Asian and younger patients with HER2‐positive GC.^67^ For taselisib, studies have shown that in patients with HER2‐negative, hormone receptor‐positive advanced breast cancer, regardless of PIK3CA mutation status, the combination of taselisib and fulvestrant has clinical activity.^68^, ^69^ In uterine serous carcinoma with PIK3CA mutations and overexpress HER2/neu, taselisib has demonstrated high activity in reducing tumour growth in vivo.^70^ In GC, the research and application of taselisib were limited. We examined the cytotoxic effects of taselisib and lapatinib in GC cell lines or organoids and observed that both drugs exhibited greater cytotoxicity compared with 5‐Fu and cisplatin. These results confirm the substantial potential of both taselisib and lapatinib in the treatment of GC.

CAFs are key contributors to the heterogeneity of the TME.^71^ An increasing number of studies have demonstrated the close interaction between CAF subpopulations and MPs. In cervical squamous cell carcinoma, immunosuppressive CAFs surrounded MP6 were triggered by TGF‐β in MP6 and enforced immune lockdown around MP6.^27^ In hepatocellular carcinoma, FAP^+^ fibroblast contributed to the formation of desert microenvironment around EMT‐HCC subtypes.^30^ Our analysis of CAF subsets confirmed the presence of six types of fibroblasts within GC tissues, among which myCAFs are closely associated with poor prognosis and immune therapy tolerance in GC. ST analysis revealed that myCAFs are enriched around MP7, forming an immune isolation zone around MP7, which may be a primary reason for the suppression of immunotherapy effects by myCAFs. Further studies revealed that myCAFs promote the transformation of MP7 phenotype by secreting RSPO3, which was known as a WNT‐signalling enhancer and promotes hyperproliferation in gastric gland.^72^ Conversely, GDF15, a member of the TGF‐β superfamily and derived from MP7, induced the aggregation of CAFs and NFs toward cancer cells through the TGFBR2 receptor, thereby promoting their transformation into the myCAFs subpopulation. Previous studies indicated that GDF15 from prostate cancer cells (PCa) stimulates bone marrow mesenchymal stem cells to differentiate into osteoblasts and enhances CCL2 expression, which attracts the tumour to bone surfaces and contributes to PCa bone metastasis.^73^ The latest clinical studies have confirmed that GDF15 inhibits T cell function and migration, and that the combination of GDF15‐neutralising antibody visugroumab significantly enhances the efficacy of the PD‐1 antibody Nivolumab in non‐squamous non‐small cell lung cancer and urothelial carcinoma.^74^ In our research, GDF15 was involved in the recruitment and switch of myCAFs, which reinforces the immuno‐isolation microenvironment around MP7 state tumour cells, providing a new perspective on the obstruction of T cell infiltration mediated by GDF15.

The present study focused on the prevalent heterogeneity states within GC and elucidated the interactions between the myCAFs subgroup and the metastasis‐associated MP MP7; however, the interaction mechanisms of other MPs with the TME warranted further investigation, especially the significance of MP3 and MP4 in tumour immunity that has not yet been fully elucidated. Notably, some null associations between certain MP and tumour progression/TME components may reflect current technical limitations in sample size rather than true biological independence. These findings specifically highlight the need for expanded validation through larger single‐cell cohorts and enhanced ST profiling. Moreover, heterogeneity and tumour‐stroma crosstalk, particularly the interaction of CAFs with tumour cells via GDF15/RSPO3, which fosters an immunosuppressive microenvironment around tumour cells, require further confirmation through additional functional experiments. Furthermore, the potential of targeting GDF15 or RSPO3 to augment immune cell penetration and increase the effectiveness of immunotherapy in patients with GC needs further investigation.

In summary, through single‐cell transcriptomics and ST, we systematically revealed robust heterogeneity in GC and preliminarily clarified the biological significance of each MP. The close interaction between MP7 and myCAFs affects the response to immunotherapy in GC, indicating that MP7 is a promising target for improving immunotherapeutic efficacy in GC. Furthermore, the prediction and verification of approved drugs that target MP7 GC cells may guide clinical treatment decisions.

## AUTHOR CONTRIBUTIONS

*Writing – original draft, visualisation, validation, methodology, investigation, formal analysis and conceptualisation*: Xiongyan Wu and Baolong Li. *Writing – review and editing, investigation, funding acquisition and conceptualisation*: Zhijian Jin. *Validation and investigation*: Yifan Lu. *Formal analysis*: Junyi Hou; Lizhong Yao and Zhenjia Yu. *Writing – review and editing and funding acquisition*: Qingqing Sang. *Methodology*: Beiqin Yu and Jianfang Li. *Resources*: Chen Li; Chao Yan and Zhenggang Zhu. *Writing – review and editing, supervision, project administration, funding acquisition and conceptualisation*: Kaiwen Tang; Bingya Liu and Liping Su.

## CONFLICT OF INTEREST STATEMENT

The authors declare no conflicts of interest.

## ETHICS STATEMENT

Our study complied with all relevant ethical regulations of Shanghai Jiao Tong University. GC patients in this study underwent radical gastrectomy at Shanghai Ruijin Hospital. All samples were obtained with the patients’ informed consent, and the samples were histologically confirmed.

## CONSENT

All authors agreed to the publication of the article to the journal.

## Supporting information




**Supplementary Figure 1. Heterogeneity within GC revealed by integrating scRNA‐seq profiles from three cohorts. A**. Distributions of counts or features per cell in each sample. **B‐C**. UMAP plot of 152,540 cells clustered by Seurat (**B**) and colored by patient (**C**). **D**. UMAP plot of all cell types in each cohort.

**Supplementary Figure 2. Comparison of pan‐cancer and GC programs. A**. Heatmap showing Jaccard similarity between MPs identified in GC and the pan‐cancer meta‐program proposed by Gavish et al. **B**. Heatmap showing Jaccard similarity between MPs identified in GC and cell type annotations from four previous GC studies. **C‐E**. UMAP plot displaying the clustering of tumor cells after integration with Harmony, colored by sample (**C**), Seurat cluster (**D**), and corresponding cell type identified by the MP signature scores (**E**), respectively. **F**. Sankey diagram illustrating the relationship between MP cell types and Seurat clusters.

**Supplementary Figure 3. Pseudotime analysis of MPs. A**. Heatmap showing the expression patterns of meta‐programs in branched expression analysis modeling. **B**. Expression dynamics of MP2 (CLDN18), MP3 (VIM), MP6 (REG1A), and MP7 (SOX4) during pseudotime progression in GC cells. **C**. The cut‐point and distribution plot used for the KM curve of MP7 in Figure 2H. **D**. Box plot showing the MP7 scores of deep and superficial primary tumors in the GSE167297 cohort. **E**. Box plot showing the MP7 scores of primary tumor and metastatic tissues in the GSE163558 cohort.

**Supplementary Figure 4. Distribution of MPs at tumor‐stromal interface A**. The delineation of tumor boundaries in spatial transcriptomics analysis. **B**. The proportion of each MP at the tumor boundary and within the tumor interior. **C**. Heatmap showing Pearson's correlation coefficients between the signature scores of each MP at the tumor‐stromal interface and the neighborhood scores of various TME cell types across 21,315 tumor spots within nine slides. **D**. Boxplot showing all cell types between the responsive (R) and non‐responsive (N) immunotherapy groups in the PRJEB25780 cohort.

**Supplementary Figure 5. MP3 as a type of epithelial cell state A**. H&E staining and spatial distribution of spots colored by cell‐type scores in representative slide 21_00731. **B**. Spatial distributions of the MP3 score, epithelial score, immunescore and CD8 T cell inflamed gene expression profile in representative slide 21_00731. **C**. IHC of CD8, FAP, RAC2 and EGR1 at MP3‐ and MP7‐dominated tumor regions. **D**. MP3 marker expression (CXCR4, RAC2 and IL‐7R) in GC cells purified from two GC tissues was detected via flow cytometry.

**Supplementary Figure 6. Correlations between MPs and fibroblast subtypes. A**. Correlation heatmap displaying the Pearson correlation coefficients between each MP signature score and the fraction of each CAF subcelltype in the TME. **B**. Heatmap showing Pearson's correlation coefficients between the signature scores of each MP and the neighborhood scores of each CAF subcelltype within nine slides. **C**. Scatter plots showing Pearson's correlation between gene signature scores of MPs and fractions of apCAFs or iCAFs across 44 GC samples. **D**. The cut‐point and distribution plot used for the KM curve of myCAFs in Figure 5G. **E**. The cut‐point, distribution plot and Kaplan‐Meier curves of iCAFs in the TCGA‐STAD cohort.

**Supplementary Figure 7. Signaling pathways enriched in myCAFs and the ligand‐receptor pairs between myCAFs and MPs. A**. Transwell assay showing the effects of conditioned medium from HGC27, NUGC3 or exogenous GDF15 on the migration ability of NFs and CAFs. **B**. PCR detection of GDF15 knockdown efficiency by siRNA. **C**. PCR assays showing the expression of TGFBR2 and GFRAL in the GC cell lines, NFs and CAFs. **D**. The expression patterns of TGFBR2 and GFRAL in the TCGA‐STAD dataset. **E**. PCR detection of TGFBR2 knockdown efficiency by siRNA. **F**. Migration assays demonstrating the effect of GDF15 on the migratory activity of NFs and CAFs after TGFBR2 knockdown. Data from three independent experiments are presented as bar graphs showing the mean values  ±  SEM. **G**. Dot plot showing significant ligand‐receptor pairs between myCAFs and meta‐programs. **H**. Dot plot showing the top enriched terms from the KEGG analysis of the top 100 DEGs of myCAFs.

**Supplementary Figure 8. Screening potential targeted drugs for MP7 using Beyondcell. A**. Survival curves of GC cells treated with escalating doses of the indicated compounds. **B**. UMAP plot showing Beyondcell's therapeutic clusters. **C**. Sankey diagram illustrating the relationship between MP cell types and Beyondcell's therapeutic clusters. **D**. Western blot of PI3K/AKT pathway‐related proteins in GC cells following EGR1 knockdown via EGR1 siRNA (siEGR1‐1).

Supporting Information

Supporting Information

Supporting Information

Supporting Information

Supporting Information

Supporting Information

## Data Availability

All the data utilised in this study are publicly accessible as outlined in the *Methods* section. The source code and proprietary cohort data used for the results can be accessed by contacting the corresponding authors for legitimate requests.
