# Supplementary figures and images for: Deciphering of intra‐tumoural heterogeneity and the interplay between metastasis‐associated meta‐program and myofibroblasts in gastric cancer
Source: Clin Transl Med. 2025 Apr 28;15(5):e70319. doi: 10.1002/ctm2.70319 (PMC12035649; doi:10.1002/ctm2.70319)

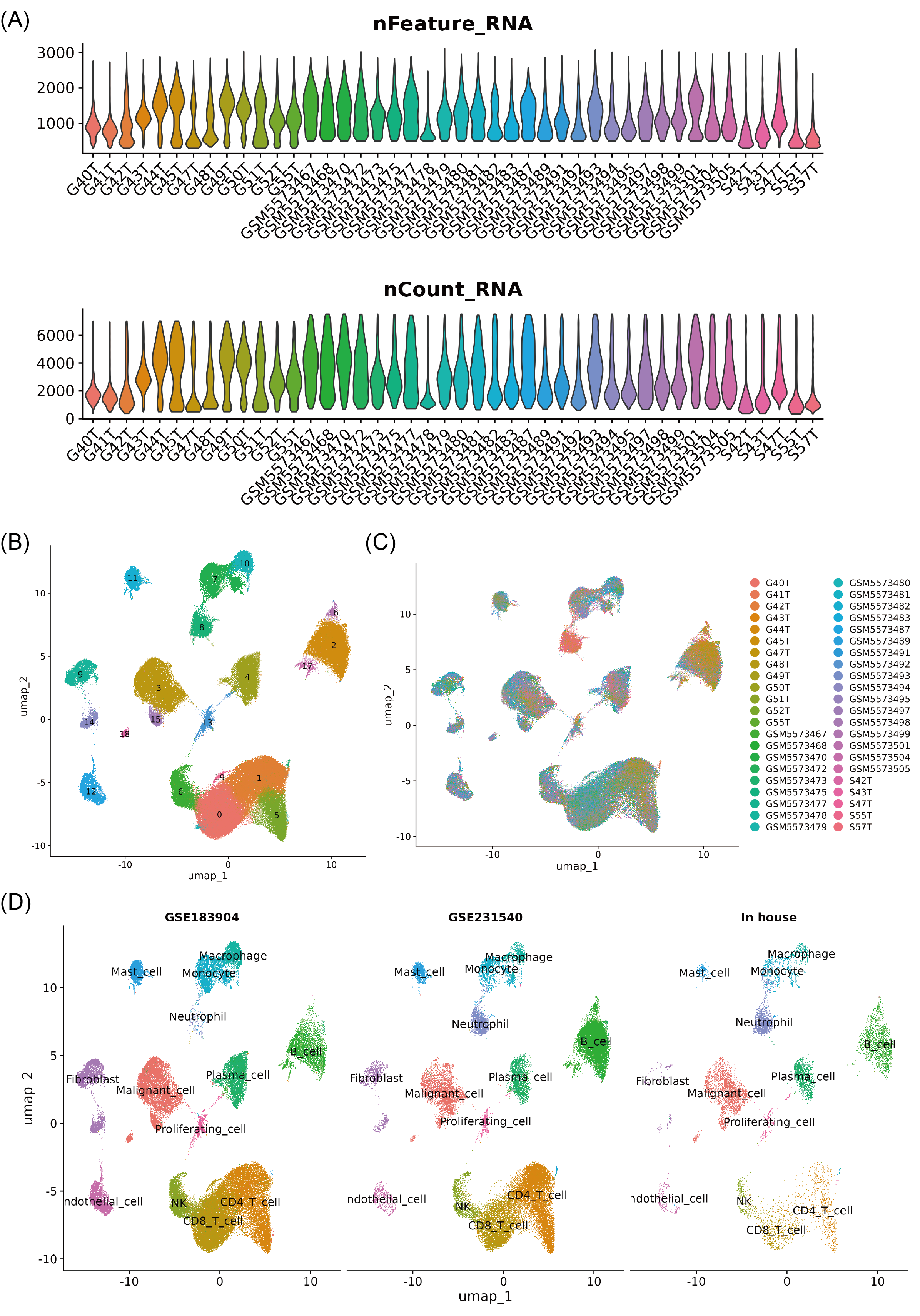

Supplement: Supplementary file 1 — Supplementary Figure 1. Heterogeneity within GC revealed by integrating scRNA‐seq profiles from three cohorts. A. Distributions of counts or features per cell in each sample. B‐C. UMAP plot of 152,540 cells clustered by Seurat (B) and colored by patient (C). D. UMAP plot of all cell types in each cohort. [file CTM2-15-e70319-s010.tif]

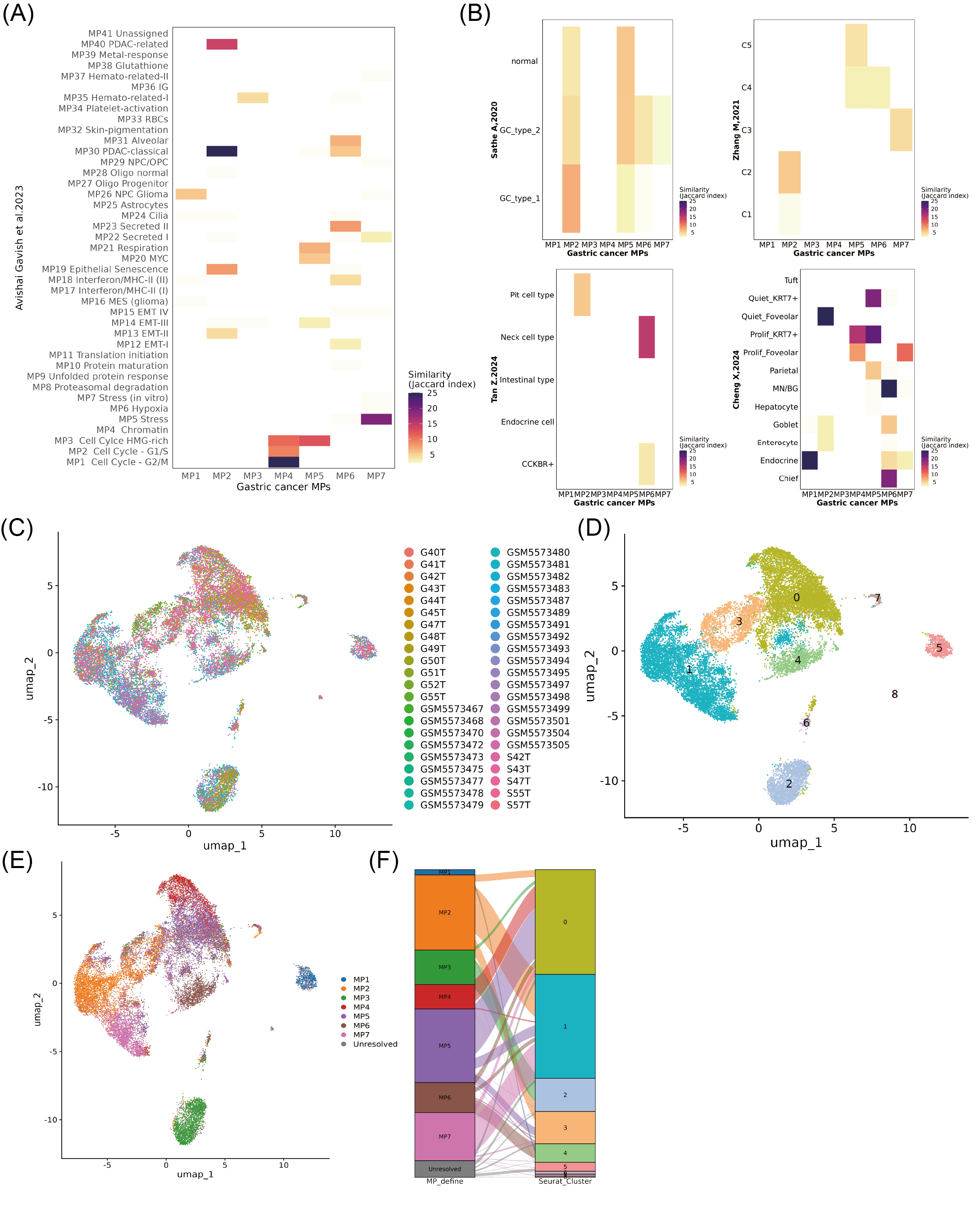

Supplement: Supplementary file 2 — Supplementary Figure 2. Comparison of pan‐cancer and GC programs. A. Heatmap showing Jaccard similarity between MPs identified in GC and the pan‐cancer meta‐program proposed by Gavish et al. B. Heatmap showing Jaccard similarity between MPs identified in GC and cell type annotations from four previous GC studies. C‐E. UMAP plot displaying the clustering of tumor cells after integration with Harmony, colored by sample (C), Seurat cluster (D), and corresponding cell type identified by the MP signature scores (E), respectively. F. Sankey diagram illustrating the relationship between MP cell types and Seurat clusters. [file CTM2-15-e70319-s012.tif]

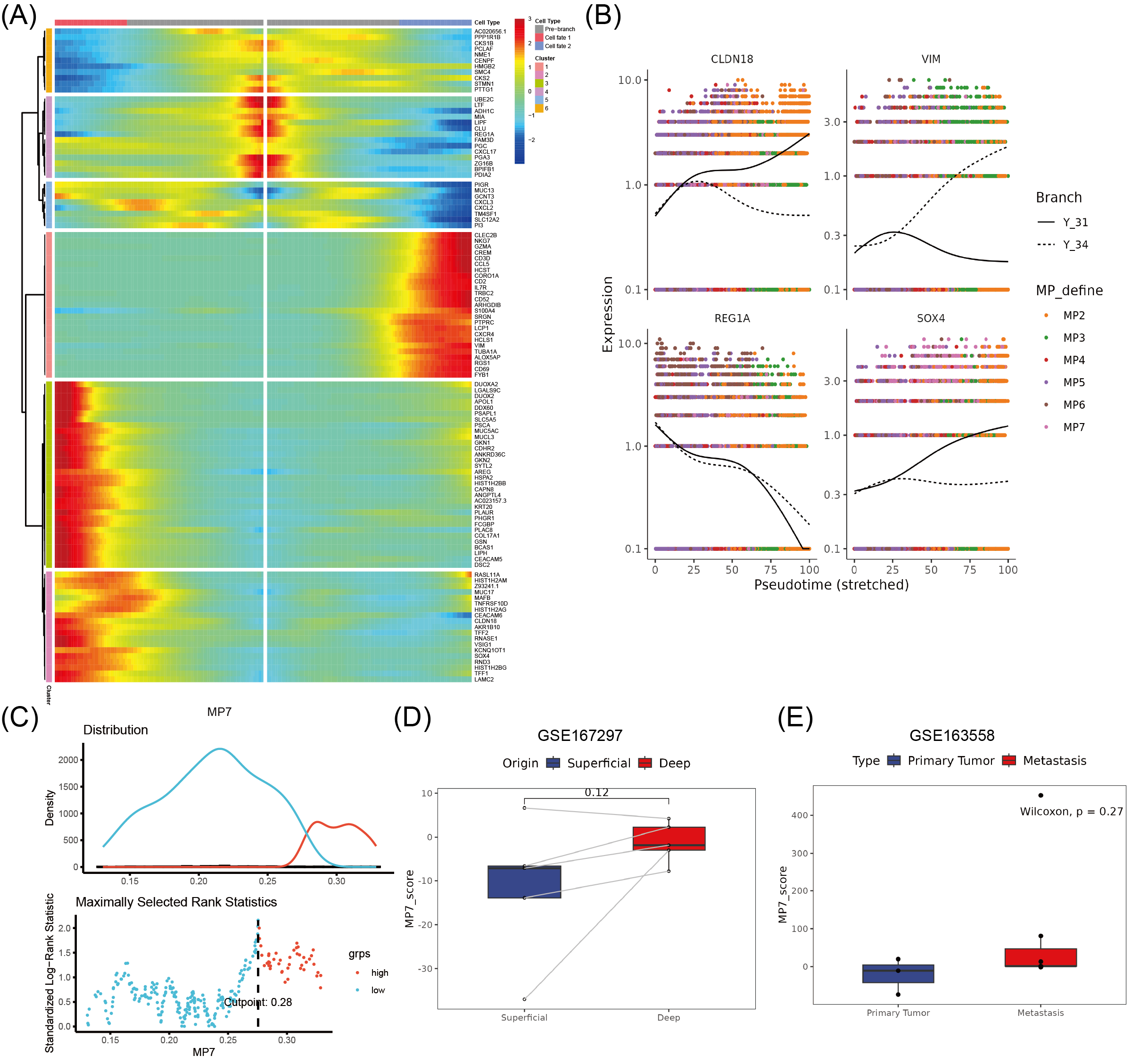

Supplement: Supplementary file 3 — Supplementary Figure 3. Pseudotime analysis of MPs. A. Heatmap showing the expression patterns of meta‐programs in branched expression analysis modeling. B. Expression dynamics of MP2 (CLDN18), MP3 (VIM), MP6 (REG1A), and MP7 (SOX4) during pseudotime progression in GC cells. C. The cut‐point and distribution plot used for the KM curve of MP7 in Figure 2H. D. Box plot showing the MP7 scores of deep and superficial primary tumors in the GSE167297 cohort. E. Box plot showing the MP7 scores of primary tumor and metastatic tissues in the GSE163558 cohort. [file CTM2-15-e70319-s005.tif]

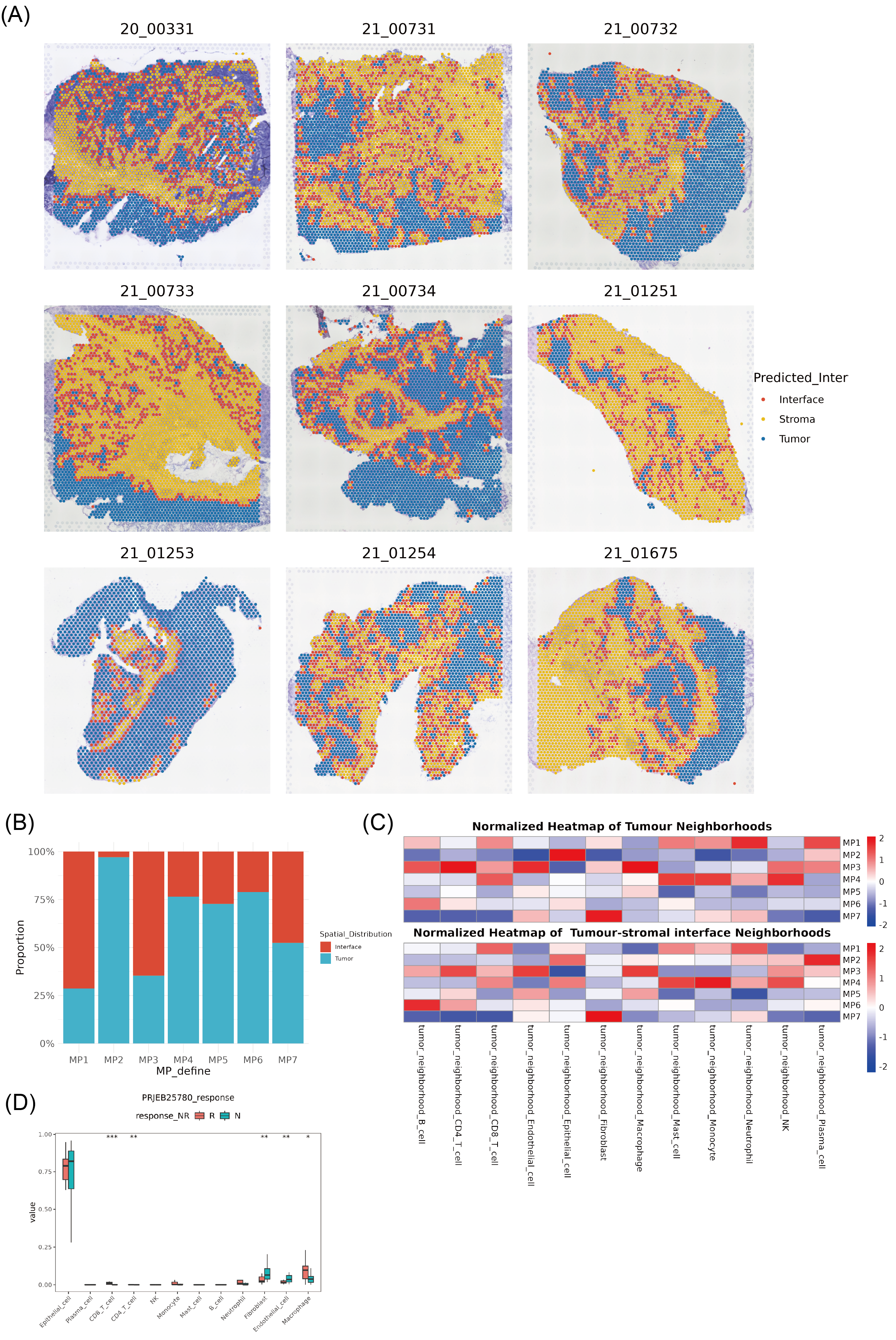

Supplement: Supplementary file 4 — Supplementary Figure 4. Distribution of MPs at tumor‐stromal interface A. The delineation of tumor boundaries in spatial transcriptomics analysis. B. The proportion of each MP at the tumor boundary and within the tumor interior. C. Heatmap showing Pearson's correlation coefficients between the signature scores of each MP at the tumor‐stromal interface and the neighborhood scores of various TME cell types across 21,315 tumor spots within nine slides. D. Boxplot showing all cell types between the responsive (R) and non‐responsive (N) immunotherapy groups in the PRJEB25780 cohort. [file CTM2-15-e70319-s008.tif]

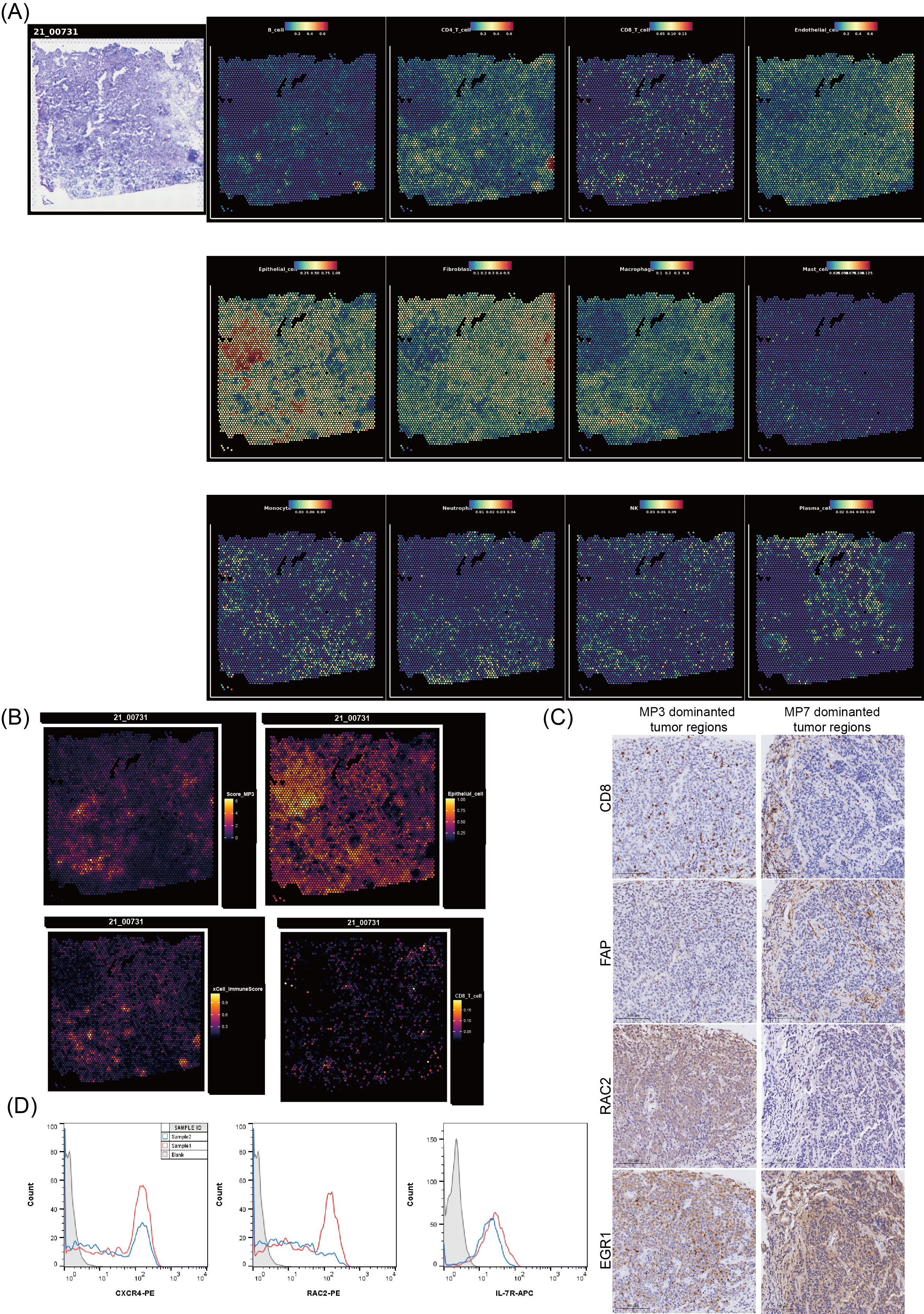

Supplement: Supplementary file 5 — Supplementary Figure 5. MP3 as a type of epithelial cell state A. H&E staining and spatial distribution of spots colored by cell‐type scores in representative slide 21_00731. B. Spatial distributions of the MP3 score, epithelial score, immunescore and CD8 T cell inflamed gene expression profile in representative slide 21_00731. C. IHC of CD8, FAP, RAC2 and EGR1 at MP3‐ and MP7‐dominated tumor regions. D. MP3 marker expression (CXCR4, RAC2 and IL‐7R) in GC cells purified from two GC tissues was detected via flow cytometry. [file CTM2-15-e70319-s001.tif]

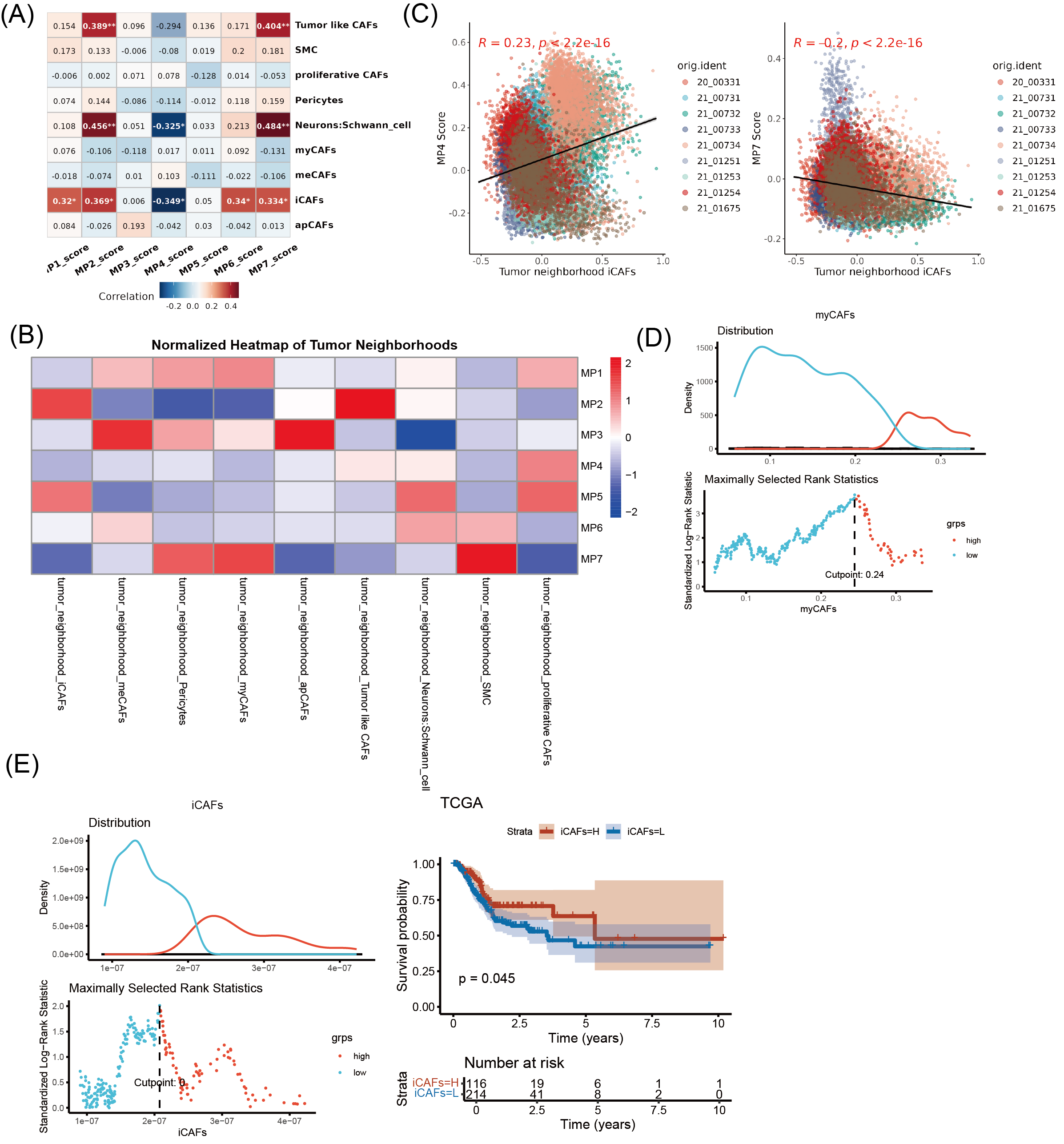

Supplement: Supplementary file 6 — Supplementary Figure 6. Correlations between MPs and fibroblast subtypes. A. Correlation heatmap displaying the Pearson correlation coefficients between each MP signature score and the fraction of each CAF subcelltype in the TME. B. Heatmap showing Pearson's correlation coefficients between the signature scores of each MP and the neighborhood scores of each CAF subcelltype within nine slides. C. Scatter plots showing Pearson's correlation between gene signature scores of MPs and fractions of apCAFs or iCAFs across 44 GC samples. D. The cut‐point and distribution plot used for the KM curve of myCAFs in Figure 5G. E. The cut‐point, distribution plot and Kaplan‐Meier curves of iCAFs in the TCGA‐STAD cohort. [file CTM2-15-e70319-s002.tif]

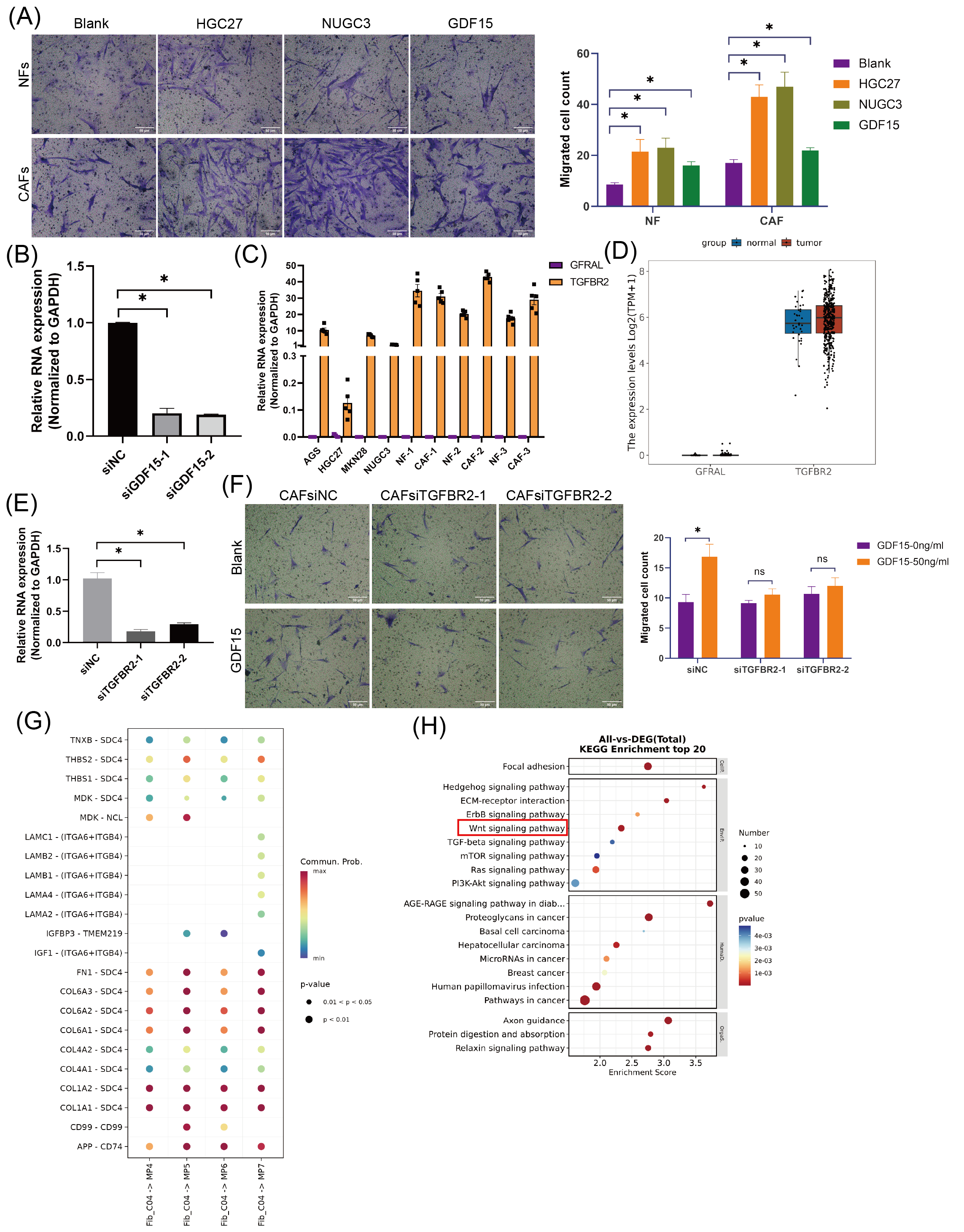

Supplement: Supplementary file 7 — Supplementary Figure 7. Signaling pathways enriched in myCAFs and the ligand‐receptor pairs between myCAFs and MPs. A. Transwell assay showing the effects of conditioned medium from HGC27, NUGC3 or exogenous GDF15 on the migration ability of NFs and CAFs. B. PCR detection of GDF15 knockdown efficiency by siRNA. C. PCR assays showing the expression of TGFBR2 and GFRAL in the GC cell lines, NFs and CAFs. D. The expression patterns of TGFBR2 and GFRAL in the TCGA‐STAD dataset. E. PCR detection of TGFBR2 knockdown efficiency by siRNA. F. Migration assays demonstrating the effect of GDF15 on the migratory activity of NFs and CAFs after TGFBR2 knockdown. Data from three independent experiments are presented as bar graphs showing the mean values ± SEM. G. Dot plot showing significant ligand‐receptor pairs between myCAFs and meta‐programs. H. Dot plot showing the top enriched terms from the KEGG analysis of the top 100 DEGs of myCAFs. [file CTM2-15-e70319-s009.tif]

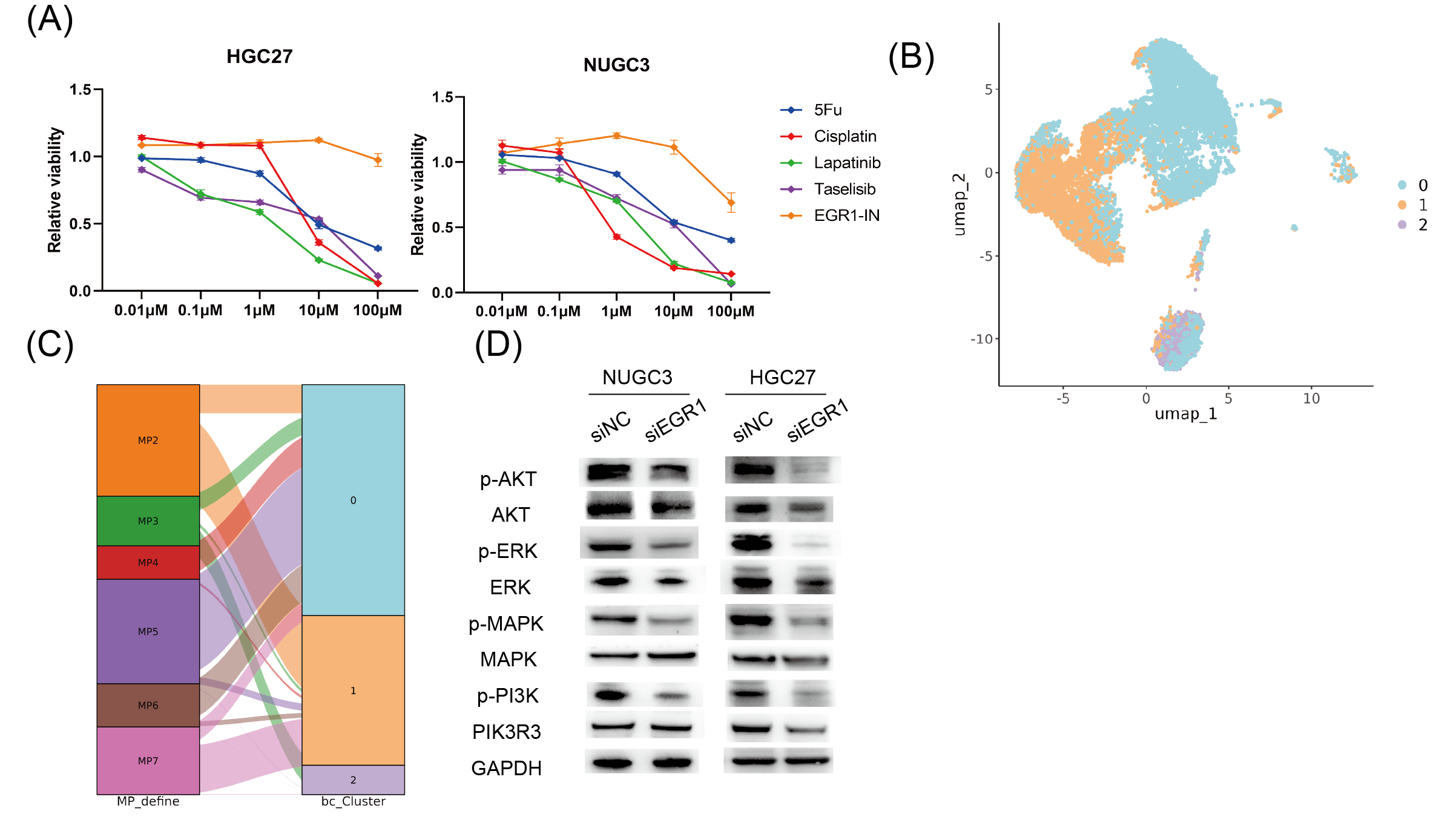

Supplement: Supplementary file 8 — Supplementary Figure 8. Screening potential targeted drugs for MP7 using Beyondcell. A. Survival curves of GC cells treated with escalating doses of the indicated compounds. B. UMAP plot showing Beyondcell's therapeutic clusters. C. Sankey diagram illustrating the relationship between MP cell types and Beyondcell's therapeutic clusters. D. Western blot of PI3K/AKT pathway‐related proteins in GC cells following EGR1 knockdown via EGR1 siRNA (siEGR1‐1). [file CTM2-15-e70319-s006.tif]
