# Supplementary material for: Deciphering of intra‐tumoural heterogeneity and the interplay between metastasis‐associated meta‐program and myofibroblasts in gastric cancer
Source: Clin Transl Med. 2025 Apr 28;15(5):e70319. doi: 10.1002/ctm2.70319 (PMC12035649; doi:10.1002/ctm2.70319)
Supplement: Supplementary file 14 — Supporting Information [file CTM2-15-e70319-s004.docx]

**Supplementary Methods**

1. **Cell lines**

The human GC cell line HGC-27 was sourced from the Cell Bank of Type Culture Collection of the Chinese Academy of Sciences, and the NUGC3 cell line was obtained from Wuhan Pricella Biotechnology Company. All the lines were verified by STR profiling and cultured in RPMI-1640 medium supplemented with 10% FBS, 100 U/mL penicillin, and 100 μg/mL streptomycin at 37°C with 5% CO2.

Fibroblasts were extracted and cultured from GC tissues following our previous methods^41,42^. The tumor tissues were cut into 3mm^3^ pieces and placed in DMEM supplemented with 10% FBS, 100 U/mL penicillin, and 100 μg/mL streptomycin. Cancer-associated fibroblasts (CAFs) were observed after 7-10 days.

1. **Isolation of** **tumor cells from GC tissue**

Tumor cells from fresh GC tissue were enriched using the EasySep™ Human EpCAM Positive Selection Kit (Stem Cell Technologies) following the manufacturer's instructions. Tissue dissociation was performed with the Tumor Dissociation Kit (Miltenyi Biotech). A 100 μL enrichment cocktail was added to 2 mL of single cell suspension, mixed, and incubated at room temperature for 5 minutes, followed by adding 100 μL of RapidSpheres. After gentle mixing, the sample was placed in an EasySep magnet for 10 minutes. The enriched cell suspension was transferred to a FACS tube.

1. **Flow cytometry**

EpCAM-positive tumor cells from GC tissues were stained with MP markers. The tumor cell suspensions were incubated with primary antibodies at room temperature for 1 hour. Unbound antibodies were washed off, and the cells were then incubated with certain secondary antibodies in the dark for 30 minutes. The antibodies and dilutions used were detailed in Supplementary Table. The cells were analyzed using a BD Calibur cytometer and FlowJo software (v10).

1. **TSA-Associated Multiplex Immunofluorescence**

Tyramide signal amplification (TSA) associated multiplex immunofluorescence was utilized to analyze MP cells and the microenvironment cell composition. Briefly, FFPE sections were processed and incubated with primary antibodies overnight at 4°C and then with HRP-conjugated secondary antibodies at 37°C for 45 minutes. TSA with specific fluorophores was applied, followed by antigen retrieval and washing. After three rounds, the sections were counterstained with DAPI and imaged using a confocal microscope (LSM510; Zeiss, Germany). Antibodies and dilutions were detailed in Supplementary Table.

1. **Patient-derived organoid (PDO)**

Patient-derived organoids (PDOs) from GC tissues were established as previous described^43^. Fresh tissues were rinsed with PBS, cut into 3-5 mm^3^ pieces, and digested with Tumor Tissue Digestion Solution (Absin) for 30 minutes. Then, the suspension was filtered through a 70 µm screen (Corning), and single cells were collected by centrifugation at 450 × g. The cell pellet was resuspended in DMEM and mixed with an equal volume of Matrigel (Corning). This Matrigel-cell mixture was plated in 24-well plates and incubated at 37°C for 30 minutes. Finally, 750 μL of human gastric cancer organoid medium (OuMel Bio) was added to each well.

1. **Gene knockdown with RNA interference (RNAi)**

The cells were seeded in six-well plates and transfected with siRNA via LipofectamineTM 3000 (Thermo Fisher) following the manufacturer's instructions. The siRNA sequences were detailed in Supplementary Table. Gene and protein expression were evaluated by qPCR and Western blotting, respectively, after transfection for 48 hours.

1. **Quantitative PCR (qPCR)**

To extract mRNA from cells and PDOs, TRIzol reagent (Solarbio) was used to lyse the cells, followed by chloroform and isopropyl alcohol for RNA concentration. Total RNA (1 μg) was reverse transcribed to cDNA with Hiscript Q-RT SuperMix (Vazyme), and qRT-PCR was conducted to quantify cDNA levels using ChamQ Universal SYBR qPCR Master Mix (Vazyme). RNA expression was normalized to GAPDH and analyzed by ΔΔCt method. GraphPad Prism 8 was used for statistical analysis, and the data were presented as the means ± SDs. The primers used were synthesized by Sangon Biotech and listed in Supplementary Table S.

1. **Western blot analysis**

The cells were lysed in RIPA buffer (Solarbio) supplemented with a protease and phosphatase inhibitor cocktail on ice for 10 minutes. Protein concentrations were measured using the BCA kit (Thermo Fisher), and 10 μg of protein was loaded per Western blot. Proteins were separated on FuturePAGE™ 4-20% gels (ACE bio) and transferred to 0.22 μm PVDF membranes (Bio-Rad). The membranes were blocked with 5% BSA in TBS-T for 1 hour, incubated with primary antibodies overnight at 4°C, and then incubated with HRP-conjugated secondary antibodies for 1 hour at room temperature. Detection was performed via a Western Blotting ECL Kit (NCM Biotech). For details and antibody information, refer to Supplementary Table.

1. **Cell viability assays**

Cell viability was assessed with the CCK-8 assay (Dojindo) following the manufacturer's protocol. Approximately GC cells or PDOs were plated and treated with various drug concentrations for 72 hours. Viability was determined by measuring absorbance at 450 nm using a microplate reader (Epoch; BioTek). Statistical analysis was performed using GraphPad Prism 8, with the data shown as the means ± SDs.

1. **Transwell Migration Assay**

Transwell assays were conducted with 8.0 µm pore polycarbonate membranes (Corning). GC cells suspended in RPMI-1640 medium were seeded in the upper chambers of 24-well plates, while complete medium was added to the lower chambers. After incubation for 24 hours, the migrated cells were fixed, stained with 1% crystal violet for 1 hour, and washed with PBS, and the non-migrated cells were removed with a cotton swab. The dried membranes were examined under a microscope at 20× magnification, and ten random fields were counted for cell migration analysis using GraphPad Prism 8. Data are presented as the means ± SDs.
